# CspZ variant–specific interaction with factor H incorporates a metal site to support Lyme borreliae complement evasion

**DOI:** 10.1016/j.jbc.2024.108083

**Published:** 2024-12-14

**Authors:** Kalvis Brangulis, Valerie Sürth, Ashley L. Marcinkiewicz, Inara Akopjana, Andris Kazaks, Janis Bogans, Alisa Huber, Yi-Pin Lin, Peter Kraiczy

**Affiliations:** 1 Latvian Biomedical Research and Study Centre, Riga, Latvia; 2 Department of Human Physiology and Biochemistry, Riga Stradins University, Riga, Latvia; 3 Goethe University Frankfurt, University Hospital of Frankfurt, Institute of Medical Microbiology and Infection Control, Frankfurt, Germany; 4 Division of Infectious Diseases, Wadsworth Center, New York State Department of Health, Albany, New York, USA; 5 Department of Infectious Disease and Global Health, Cummings School of Veterinary Medicine, Tufts University, North Grafton, Massachusetts, USA; 6 Department of Biomedical Sciences, SUNY Albany, Albany, New York, USA

**Keywords:** Lyme disease, spirochete, *Borrelia*, complement, factor H, bacterial pathogenesis, zinc

## Abstract

Polymorphic microbial immune evasion proteins dictate the pathogen species- or strain-specific virulence. Metals can impact how microbial proteins confer host–pathogen interactions, but whether this activity can be allelically variable is unclear. Here, we investigate the polymorphic CspZ protein of Lyme disease spirochete bacteria to assess the role of metals in protein–protein interaction. CspZ facilitates evasion of the complement system, the first line of immune defense through binding to the complement regulator factor H (FH). By obtaining a high-resolution cocrystal CspZ–FH structure, we identified a zinc coordinating the binding of FH SCR6–7 domains to a Glu65 on a loop from CspZ of *Borrelia burgdorferi* B31. However, zinc is dispensable for human FH binding for CspZ orthologs with a different loop orientation and/or lacking this glutamate. Phylogenetic analysis of all known human FH–binding CspZ variants further grouped the proteins into three unique lineages correlating with loop sequences. This suggests multiple FH-binding mechanisms evolved through Lyme disease spirochete–host interactions. Overall, this multidisciplinary work elucidates how the allelically specific immune evasion role of metals is impacted by microbial protein polymorphisms.

Protein polymorphism is present in many pathogenic microorganisms, reflecting the diverse infection phenotypes of those pathogens during their interactions with hosts (1). For human pathogenic microorganisms, polymorphic microbial proteins that promote immune evasion are a contributing factor of different infection outcomes and disease severity (2, 3). However, the molecular basis dictating such polymorphism-mediated infection phenotypes are not fully demonstrated. Metal ions play essential roles in modulating the viability of many pathogens (4). The concept of “nutritional immunity,” in which hosts can limit the amount of metal ions available for pathogens to use to control the infectivity, underscores pathogens’ critical needs of metal ions (5). Transition metals (*e.g.*, iron [Fe], copper [Cu], and zinc [Zn]) modulate the stability and functions of microbial proteins, including immune evasion determinants (6). Several recent observations showed some proteins of human pathogenic bacteria that require a metal ion for their inherent infection-related functions are polymorphic (7, 8, 9). These findings lead to a possibility that genetically polymorphic microbial factors that require metal ions to confer immune evasion differ in the extents of the need for the metal ions to promote immune evasion functions.

Lyme disease is the most prevalent vector-borne disease in the Northern hemisphere (10). Transmitted by *Ixodes* ticks, this human disease is caused by spirochetes that belong to multiple species within the *Borrelia burgdorferi* sensu lato (s.l.) complex (also known as *Borreliella burgdorferi*, *B. burgdorferi* s.l., or Lyme borreliae) (11). These bacterial species include numerous genetically distinct human infectious strains of North American– and Eurasian-prevalent *B. burgdorferi* sensu stricto (hereafter *B. burgdorferi*) and Eurasian-prevalent *Borrelia afzelii* and *Borrelia garinii* (11). In nature, Lyme borreliae can be carried by ticks and different vertebrate reservoir animals and infect the incidental hosts, such as humans (11). Upon transmission, Lyme borreliae disseminate from tick bite sites of the skin to distal organs. In humans, such dissemination can cause systemic manifestations, including arthritis, carditis, and neurological symptoms (12). Patients infected with different Lyme borreliae strains and/or species have varying severity of disseminated manifestations (13, 14). This is consistent with a recent report showing significant genetic diversity of the human-isolated strains or species of *B. burgdorferi* s.l (15). In addition, Lyme borreliae virulence and infectivity can be controlled by metal ions and bacterial metal-binding proteins (16, 17, 18, 19). These findings suggest that Lyme borreliae can be utilized as a model to examine how metal ions impact strain-specific infection-related phenotypes.

The efficiency of Lyme borreliae infection can be modulated by host immune responses, including complement, the first-line host immune defense (20, 21, 22). The three canonical pathways of complement are directly activated on the bacterial surface *via* (1) the interactions of microbial antigens and host antibodies (classical pathway); (2) microbial carbohydrate and host lectin (lectin pathway); and/or (3) microbial surface structure and host-derived activated C3b (alternative pathway) (23). The activation of each of these three pathways leads to opsonization, inflammation, phagocytosis, and finally pathogen lysis (23). Lyme borreliae escape complement-mediated killing and achieve disseminated infection by producing a range of outer surface proteins that bind and recruit host complement regulators or complement proteins (20, 21, 22). One group of such complement regulator-acquiring surface proteins (CRASPs) bind to the complement regulator, factor H (FH) (24). Among the five CRASPs, CspZ (CRASP-2) is predominately produced *in vivo* and facilitates *B. burgdorferi* dissemination to distal organs of vertebrate animals (25, 26, 27). Further, polymorphism of CspZ variants is linked to the FH-binding activity and the efficiency of *B. burgdorferi* strains to cause systemic infection in a host-specific manner (27, 28). Together with the aforementioned possibility that metal ions regulate strain-specific infection-related phenotypes (7, 8, 9), these results raise the question whether metal ions play a role in modulating CspZ-mediated complement evasion. If so, the need of metals for CspZ-specific phenotypes may be variant specific.

In this study, we provide a model to test the role of metals in conferring microbial protein variant-specific immune evasion. We obtained the high-resolution crystal structure of the complex formed by human FH and CspZ from *B. burgdorferi* B31, revealing the role of zinc in CspZ–FH interactions. Along with structural and functional comparisons of CspZ variants with different human FH–binding abilities, we examined the allelic-specific role of zinc in impacting protein–protein interaction. We further performed the phylogenetic analysis of all known human FH–binding CspZ variants, elucidating the evolutionary history of this genetically polymorphic zinc binding–mediated microbial immune evasion protein.

## Results

### CspZ variants display variable human FH–binding activity and confer alternative pathway evasion

**Figure 1:**
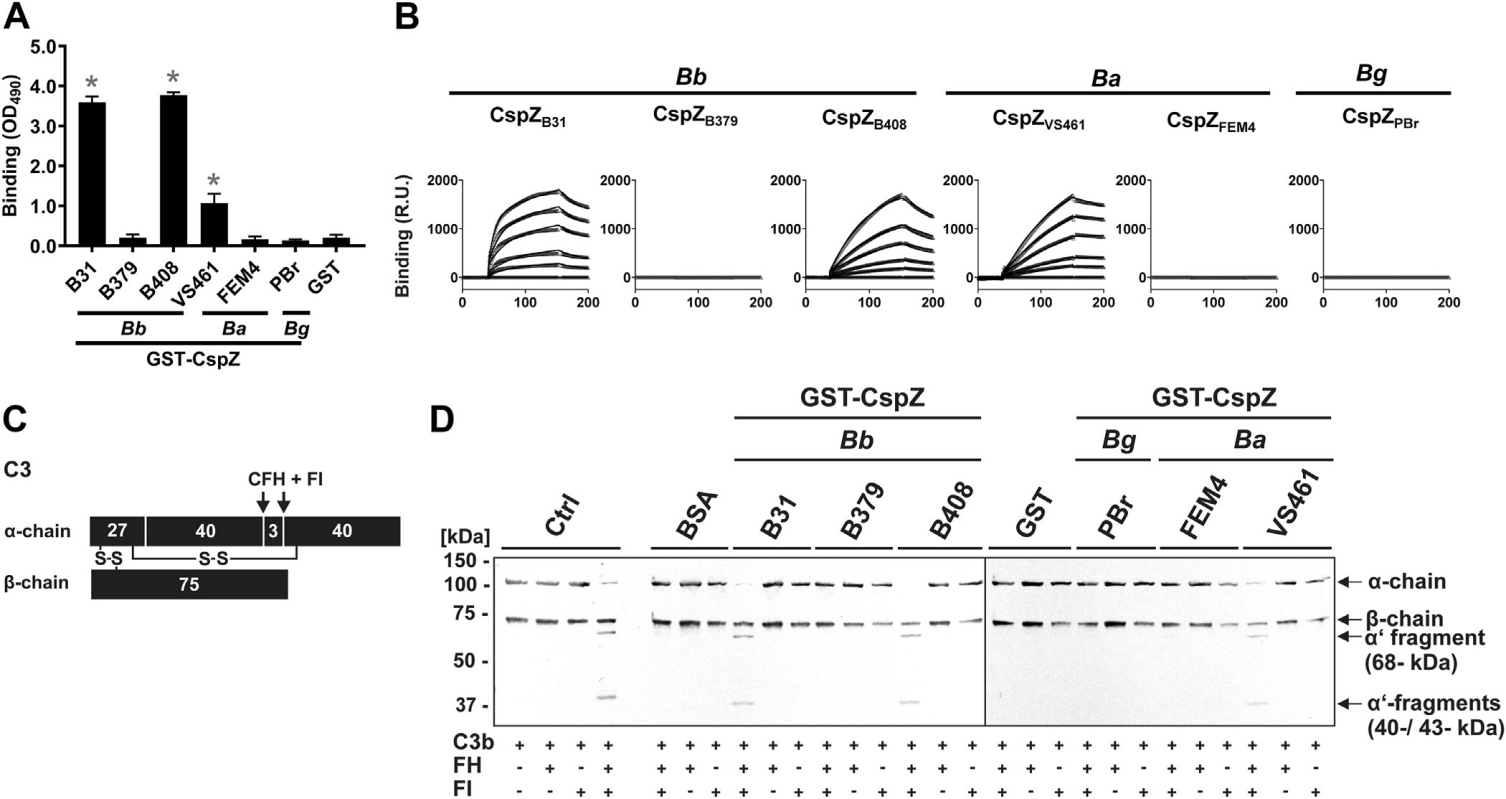
**Recombinant CspZ bound to human factor H (FH) and inactivated human alternative complement pathway in a variant-specific manner.***A*, recombinant GST-tagged proteins and GST (5 ng/μl each) were immobilized and incubated with 5 ng/μl of purified FH. Protein complexes were detected using an anti-FH antibody (1:1000 dilution). Data represent means and standard deviation of at least three independent experiments, each conducted in duplicate. ∗*p* ≤ 0.0001, ns, no statistical significance, one-way ANOVA with post hoc Bonferroni multiple comparison test (confidence interval = 95%). *B*, the indicated untagged CspZ variants from *Borrelia burgdorferi* (*Bb*), *Borrelia afzelii* (*Ba*), or *Borrelia garinii* (*Bg*) were flowed in PBS buffer over the chip surface, conjugated with human FH. Binding was measured in response units (R.U.) by SPR. The *k*_on_, *k*_off_, and *K*_D_ values were determined from the average of three experiments (Table 1). Shown is one representative experiment. *C*, schematic representation showing the disulfide-linked α-, β-, and the γ-chains of C3b, with molecular weight presented, and the sites of α-chain cleavage by FH and FI indicated by *arrows*. *D*, microtiter plates coated in triplicate with BSA (“Ctrl,” control), GST, or purified GST-tagged CspZ proteins (1 μg each) were incubated with FH (1 μg) (two wells) or without FH (one well). Reaction mixtures only containing C3b (10 μg/μl) plus FI (20 μg/μl) or C3b were also included. Supernatants from the ELISA plate wells were immunoblotted with polyclonal anti-C3 antibody to detect cleavage products. BSA, bovine serum albumin; FI, factor I; GST, glutathione-*S*-transferase; SPR, surface plasmon resonance.

We first tested the binding activity of human FH to diverse CspZ variants from *B. burgdorferi*, *B. afzelii*, and *B. garinii*. CspZ variants from *B. burgdorferi* B31 and B408 (CspZ_B31_ and CspZ_B408_, respectively), but not the variant from B379 (CspZ_B379_), had significantly greater human FH binding than glutathione-*S*-transferase (GST) used as a control protein (Fig. 1*A*). This was consistent with detectable and indistinguishable human FH–binding affinity values (*K*_D_) of CspZ_B31_ and CspZ_B408_, but not CspZ_B379_, determined by surface plasmon resonance (SPR) (Fig. 1*B* and Table 1). Between the CspZ variants from two *B. afzelii* strains analyzed, CspZ from VS461 (CspZ_VS461_), but not CspZ from FEM4 (CspZ_FEM4_), yielded human FH–binding affinity significantly greater than the GST control protein (Fig. 1, *A* and *B*, and Table 1). In contrast, the CspZ variant from *B. garinii* strain PBr (CspZ_PBr_) did not bind to human FH (Fig. 1, *A* and *B*, and Table 1). In addition to recombinant FH, human FH–binding variants (*i.e.*, CspZ_B31_ and CspZ_B408_) bound FH from human sera (Fig. S1).

**Table 1:** The human factor H-binding affinity of CspZ variants in the presence and absence of EDTA and zinc

CspZ variant	Treatment	Surface plasmon resonance^a^		
		*K*_D_ (μM)	*k*_on_ (10^3^s^−1^ M^−1^)	*k*_off_ (s^−1^)
CspZ_B31_	PBS	0.34 ± 0.20	66.63 ± 5.22	0.021 ± 0.012
	EDTA	3.05 ± 5.92^b^	4.37 ± 2.37^b^	0.012 ± 0.004^b^
	EDTA–Zn	0.68 ± 0.83	51.23 ± 7.11	0.035 ± 0.008
CspZ_B379_	PBS	NB^c^	NB	NB
	EDTA	ND^d^	ND	ND
	EDTA–Zn	ND	ND	ND
CspZ_B408_	PBS	0.54 ± 0.27	14.13 ± 1.47	0.0079 ± 0.0048
	EDTA	0.49 ± 0.27	13.93 ± 1.45	0.0071 ± 0.0046
	EDTA–Zn	ND	ND	ND
CspZ_VS461_	PBS	0.37 ± 0.29	9.42 ± 1.34	0.0034 ± 0.0016
	EDTA	0.47 ± 0.25	10.58 ± 3.09	0.0045 ± 0.0016
	EDTA–Zn	ND	ND	ND
CspZ_FEM4_	PBS	NB	NB	NB
	EDTA	ND	ND	ND
	EDTA–Zn	ND	ND	ND
CspZ_PBr_	PBS	NB	NB	NB
	EDTA	ND	ND	ND
	EDTA–Zn	ND	ND	ND

All values represent the mean ± standard deviation of three experiments.

a Determined using untagged CspZ proteins.

b Because the binding of EDTA-treated CspZ_B31_ to human factor H is weak, the determined values should be considered estimates.

c No binding activity was detected.

d Not determined.

Next, we examined the ability of these CspZ variants to confer C3b degradation, an alternative pathway–inactivating mechanism dependent on the presence of FH and factor I (FI) (Fig. S2). Degradation of C3b was mediated by FH and FI in the presence of a human FH–binding control protein, resulting in the appearance of characteristic 67, 43, and 40 kDa fragments (Fig. 1*C*). We observed such an FH and FI-mediated C3b degradation pattern in the presence of CspZ_B31_, CspZ_B408_, or CspZ_VS461_ (Fig. 1*D*). In contrast, no C3b degradation could be detected with CspZ_FEM4_ or CspZ_PBr_, similar to the bovine serum albumin (BSA) and GST-containing control reactions (Fig. 1*D*). These results demonstrated CspZ allelically variable FH-binding activity and alternative pathway inactivation.

### Ectopically produced CspZ confers variant-specific human FH binding and evasion to human complement–mediated killing

**Figure 2:**
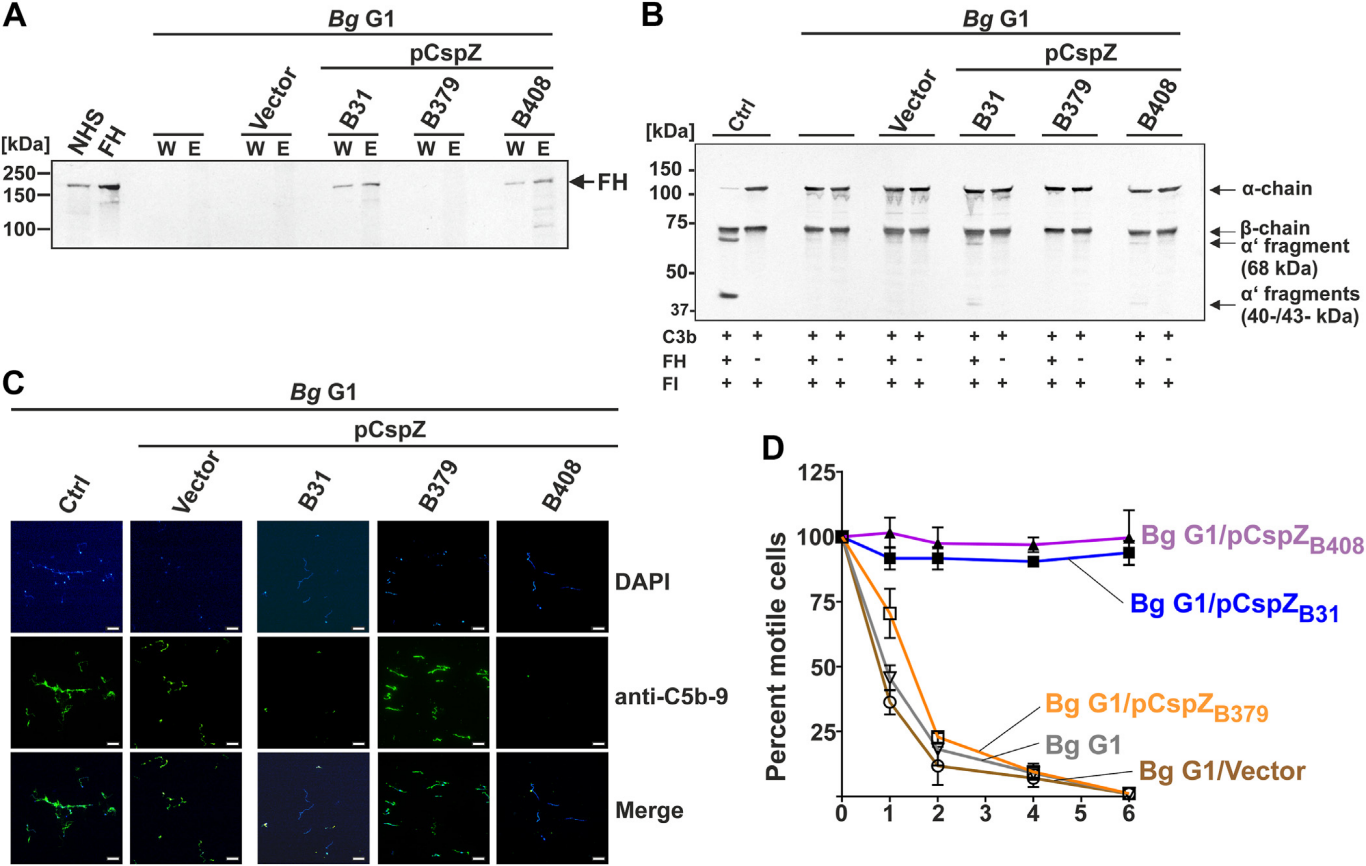
**The surface exposed CspZ promoted a variant-specific *Borrelia burgdorferi* complement inactivation and survivability in human sera.***A*, *Borrelia garinii* strain G1, or the transformants carrying an empty vector or producing each of the indicated CspZ variants, were incubated in NHS. Following serum incubation, spirochetes were washed extensively, and surface-bound proteins were eluted. Both the last wash (“W”) and the eluate (“E”) fractions obtained from each reaction were separated by 10% Tris–tricine SDS-PAGE under nonreducing conditions and transferred to Western blotting. Human FH was detected using a polyclonal anti-FH antibody. Purified human FH (500 ng) and NHS were included as controls. The location reflecting the molecular weights equivalent to FH are shown by *arrows*. *B*, immobilized *B. garinii* G1 or each of the aforementioned transformants was incubated with or without FH (5 ng/μl), followed by the incubation with C3b (6 ng/μl) and/or FI (3 ng/μl). Controls on the *left* indicated the reaction with no *B. garinii* G1-derived strains. Supernatants from the reactions were immunoblotted with polyclonal anti-C3 antibody to detect C3b cleaved products (indicated by *arrows* at *right*). *C*, indicated *B. garinii* G1-derived strains (6 × 10^6^) were incubated with 25% NHS. Deposition of activated C5b-9 complex on spirochetal surface was detected using anti-human C5b-9 antibodies (1:70 dilution) and Alexa Fluor 488-conjugated secondary antibodies (1:1000 dilution). For visualization of the spirochetes in a given microscopic field, the DNA-binding dye 4,6-diamidino-2-phenylindole (DAPI) was used. The spirochetes were observed at a magnification of 100x (bar represents 16 μm). The data were recorded with an Axio Imager M2 fluorescence microscope (Zeiss) equipped with a Spot RT3 camera (Visitron Systems). Each panel is representative of 20 microscope fields. *D*, indicated *B. garinii* G1-derived strains (4 × 10^7^) were incubated with 50% of NHS for 0, 1, 2, 4, and 6 h. Percentage of motile bacteria were counted based on the number of motile bacteria at indicated time points and normalized to counts prior to the treatment. Shown is the mean ± standard deviation of percent motile bacteria from three independent experiments. FH, factor H; NHS, nonimmune human serum.

We used CspZ variants from *B. burgdorferi* as a model to examine whether CspZ produced on the spirochete surface promotes allelically variable human FH–binding activity. Native CspZ_B31_, CspZ_B379_, or CspZ_B408_ was ectopically produced on the surface of *B. garinii* G1, a background strain that cannot bind human FH and thus is highly vulnerable to human complement–mediated killing (29). We have previously shown that these gain-of-function strains produce indistinguishable levels of CspZ_B31_, CspZ_B379_, or CspZ_B408_ on the spirochetal surface (30), confirmed herein by the digestion of CspZ under the treatment of trypsin or proteinase K *in situ* (Fig. S3). G1 producing CspZ_B31_ or CspZ_B408_, but not CspZ_B379_ or harboring the empty shuttle vector, bound FH from human serum (Fig. 2*A*), agreeing with previous results using recombinant FH (29). These collective results exhaustively demonstrated that CspZ variants show allelic specific human FH–binding ability.

After incubating each of the aforementioned G1-derived strains with purified FH, only G1 strains producing CspZ_B31_ or CspZ_B408_, but not the G1 strain producing CspZ_B379_ or carrying the empty vector, yielded the expected fragments from C3b degradation (*i.e.*, 67, 43, and 40 kDa fragments, Fig. 2*B*). We then detected deposition of the C5b-9 complex (or membrane attack complex) on each of the G1-derived strains, another proxy of complement activation. We observed human C5b-9 deposition on G1 carrying the empty vector or G1 producing CspZ_B379_, whereas G1 producing CspZ_B31_ or CspZ_B408_ cells stained negative (Fig. 2*C*). Finally, we examined if CspZ allelic-specific human complement inactivation can be extended to the ability of these G1-derived strains to survive in 50% of the human sera. After incubating each of these strains with human sera, 100% of CspZ_B31_- and CspZ_B408_-producing strains survived throughout the experiments (Fig. 2*D*). In contrast, the survival of CspZ_B379_-producing strain, the empty vector harboring strain, and the wildtype G1 strain declined continuously throughout the experiment (from 30% to 60% after 1 h of incubation to 1–2% after 6 h of incubation) (Fig. 2*D*). Overall, these results indicated the variant-to-variant differing ability of surface-produced CspZ to confer Lyme borreliae binding to human FH and the evasion of human complement–mediated killing.

### The CspZ_B31_–SCR6-7 complex display a unique zinc-binding pocket coordinated by CspZ_B31_ and bivalent FH

**Figure 3:**
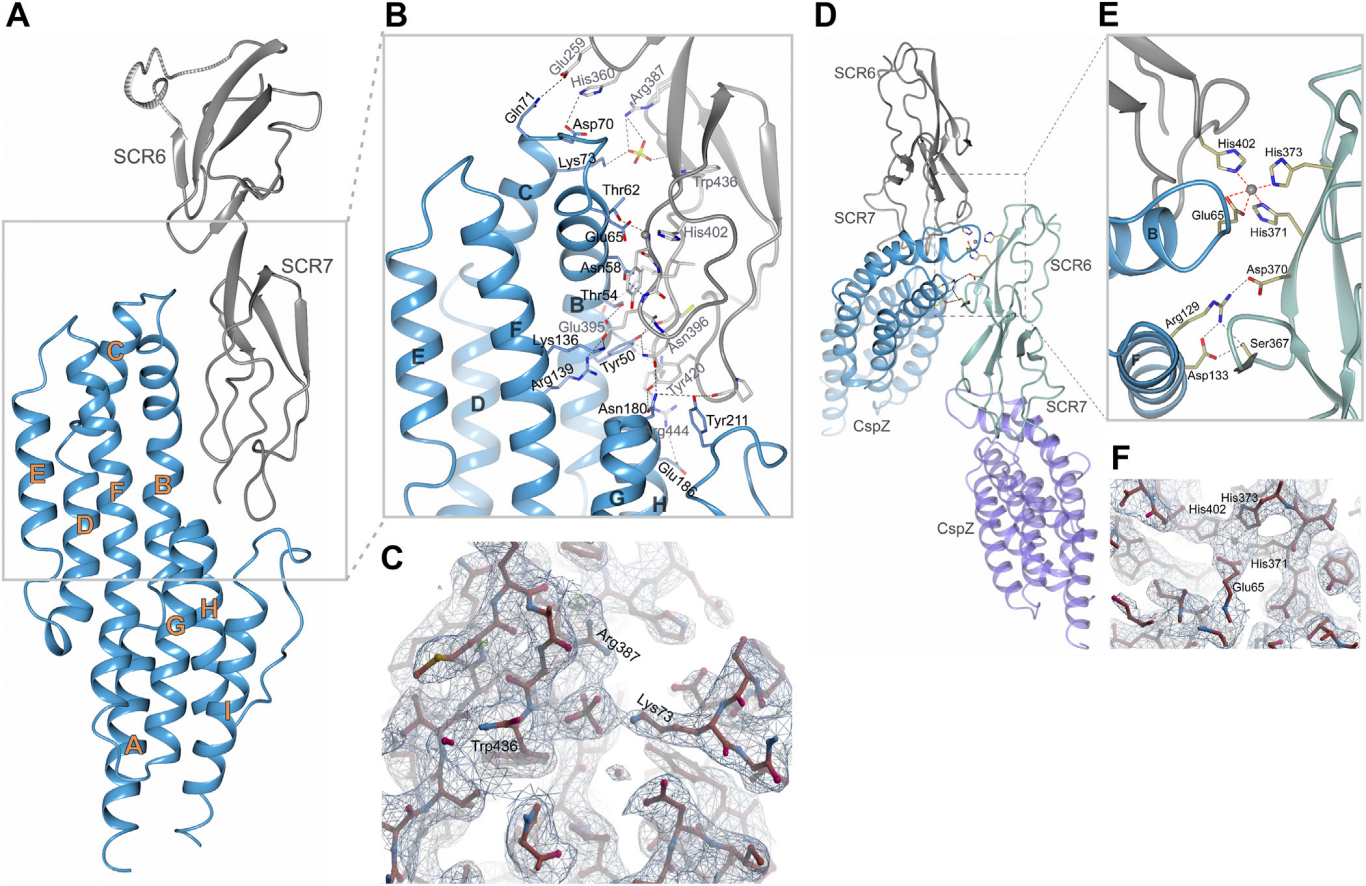
**The three-dimensional structure of the CspZ**_**B31**_**and human SCR6-7 complex, revealed by X-ray crystallography, showed a zinc-coordinated human factor H (FH)-binding mode.***A*, shown is the overall structure of CspZ_B31_–SCR6-7 complex with CspZ and SCR6-7 illustrated in *blue* and *gray*, respectively. CspZ α-helices A to I are indicated, and the striped segment in SCR6 represents residues 337 to 345 in FH that were not modeled in the final structure. *B*, the FH-binding interface of CspZ_B31_–SCR6-7 complex. CspZ_B31_ α-helices B, C, F, G, and H, along with the loops between helices B/C and H/I are involved in the FH binding of CspZ_B31_ and human SCR6-7. The amino acids on CspZ_B31_ and those on SCR6-7 involved in SCR6-7 binding are indicated in *black* and *gray fonts*, respectively. The sulfate and zinc are highlighted in *yellow/red sticks* and a *gray sphere*, respectively. *C*, the 2Fo–Fc electron density map, contoured at 1σ, represented the FH-binding interface that allows sulfate to coordinate the CspZ_B31_ and SCR6-7 interaction. *D*, CspZ_B31_–SCR6-7 complex interaction with the other SCR6-7 molecule as found in the protein crystal. *E*, the interaction interface between CspZ_B31_ and SCR6-7 complex with SCR6 from the other SCR6-7 molecule. The residues involved in coordinating zinc and those are indicated. *F*, the 2Fo–Fc electron density map, contoured at 1σ, showed zinc coordination by CspZ, SCR6, and SCR7.

We previously cocrystallized CspZ_B408_ with the SCR6-7 domain of human FH and identified the CspZ residues involved in FH binding, which are located in helices B, F, G, and loops B/C and H/I (27). Some of those equivalent residues were either missing or replaced by alternative residues in other human FH–binding CspZ variants (*i.e.*, CspZ_B31_ or CspZ_VS461_) (Fig. S4). These findings suggest that the mechanisms underlying human FH–binding ability may vary between different variants. Thus, we cocrystallized CspZ_B31_ with human SCR6-7 and obtained crystals of the CspZ_B31_–SCR6-7 complex, which diffracted to a resolution of 2.85 Å with a single copy in the asymmetric unit (Fig. 3*A*). In the CspZ_B31_–SCR6-7 complex, all residues of CspZ_B31_ were modeled in the electron density. In addition, the N-terminal residues (Gly-Ala-Met-Gly), which remained after the 6xHis tag cleavage, were built. Note that the first three residues (321–323) and residues 337 to 345 of SCR6-7 were not included in the final model because of weak electron density.

The overall structure of CspZ_B31_ possessed the previously characterized, all-α-helical fold made of nine α-helices (A–I), which is a feature distinct from other FH-binding proteins both in *B. burgdorferi* and other bacteria (Fig. 3*A*) (31). The interface between CspZ_B31_ and SCR6-7 covered an extensive 900 Å area (Fig. 3*B*). Helix B and the adjacent loop B/C of CspZ_B31_ played a major role in complex formation, involving a total of eight residues (Tyr50, Asn51, Thr54, Asn58, Thr62, Glu65, Asp70, and Gln71) (Fig. 3*B*). In addition, the residues Lys73 from α-helix C, Lys136 and Arg139 from α-helix F, Asn180 from α-helix G, Glu186 from α-helix H, and Tyr211 from the loop H/I all contributed to the complex formation (Fig. 3*B*). For SCR6-7, residues Glu259 and His360 from SCR6 interacted with residues Gln71 and Asp70 of CspZ_B31_, respectively (Fig. 3*B*). In SCR7, residues Glu395 and Arg444 were involved in interactions with at least two residues in CspZ_B31_ (Fig. 3*B*). We also found that CspZ_B31_ bound not only to both SCR6 and SCR7 from one SCR6-7 but also to SCR6 from a second FH SCR6-7 molecule (Fig. 3*D*). This additional interaction was the result of Ser367 and Asp370 of the second SCR6 binding with CspZ residues Asp133 and Arg129, respectively (Fig. 3*E*).

We found a sulfate ion to connect the residue Lys73 in CspZ_B31_ and the residues Arg387 and Trp436 in SCR7 (Fig. 3, *B* and *C*). However, as the precipitation solution used for crystallization contained sulfate, this sulfate-mediated CspZ–FH interaction was likely artificial. Furthermore, we observed a zinc ion to consistently coordinate a metal binding pocket, consisting of Glu65 of CspZ_B31_, His402 of SCR7 from one SCR6-7, and His371 and His373 of SCR6 from the second SCR6-7 (Fig. 3, *E* and *F*). The presence of zinc in the CspZ_B31_–SCR6-7 crystal was further supported by the X-ray fluorescence analysis performed on that crystal using the MX beamline instrument BL14.1 (32), revealing a major peak corresponding to the zinc atom (Fig. S5*A*). In addition, an X-ray absorption scan was performed to measure absorption around the Zn *K*-edge, showing a value characteristic of the X-ray absorption edge energy for zinc (9.6586 keV) (Fig. S5, *B* and *C*). Together with the fact that zinc was neither utilized in any purification steps nor the precipitation solution, our structural evidence indicated that environmental zinc ions coordinated the bivalent binding of FH to CspZ_B31_.

### The loop B/C-dictated structural differences determine an allelic-specific role of zinc in CspZ–FH interactions

**Figure 4:**
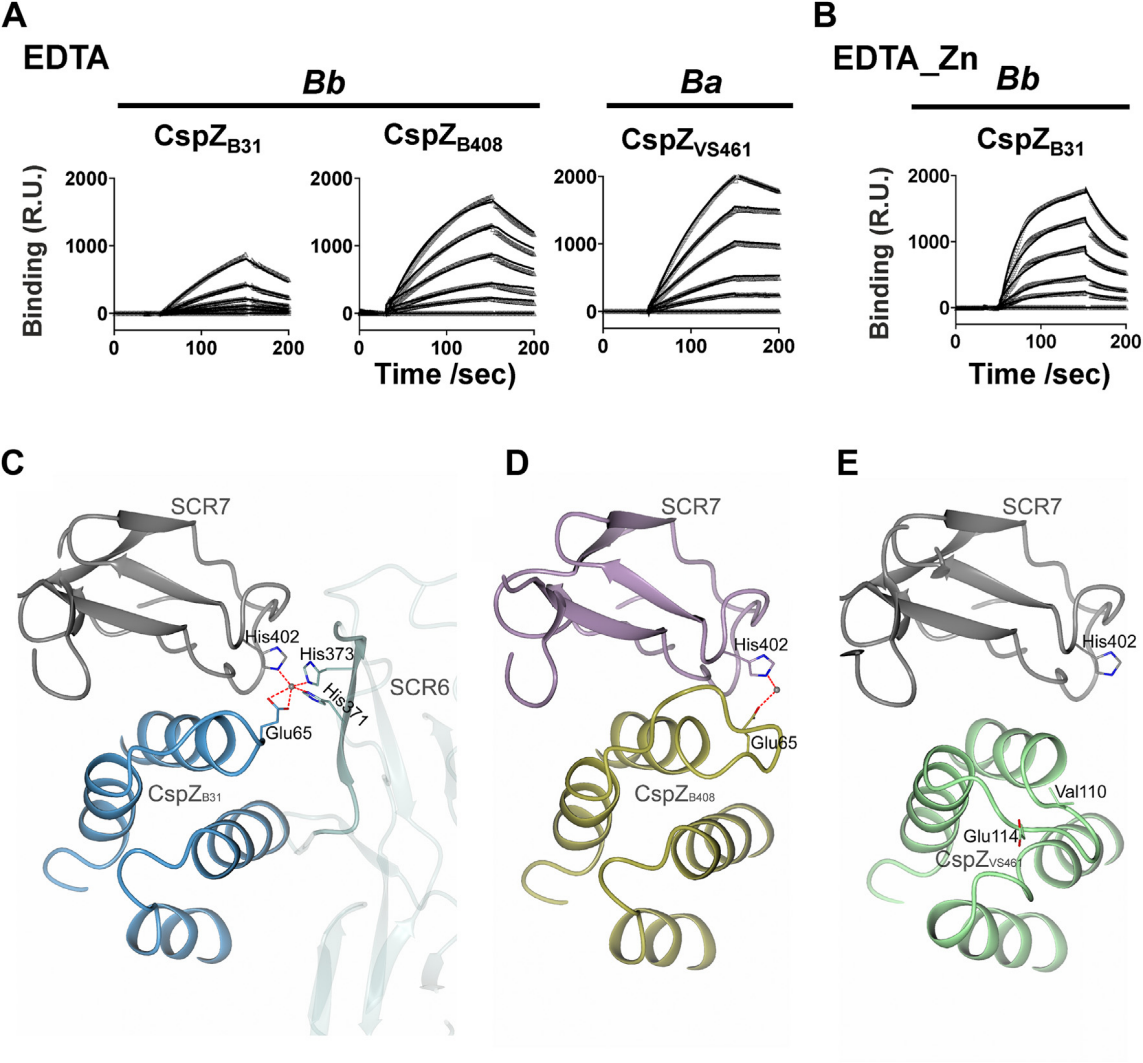
**The loop B/C polymorphisms of CspZ variants and involvement of bivalent SCR6-7 determined the role of zinc in the CspZ human factor H (FH)-binding mode.***A* and *B*, the indicated untagged CspZ variants from *Borrelia burgdorferi* (“*Bb*”) or *Borrelia afzelii* (“*Ba”*) were treated with (*A*) 100 μM of EDTA (“EDTA”), followed by dialysis with PBS buffer, and (*B*) the EDTA-treated, PBS-dialyzed CspZ_B31_ was subsequently treated 100 μM of zinc sulfate (“EDTA-Zn”). The indicated CspZ variants under each of the treatment were then flowed in PBS buffer over the chip surface, conjugated with indicated human FH. Shown are the representative results of binding measured in response units (R.U.) by SPR from one of three experiments. The *k*_on_, *k*_off_, and *K*_D_ values were determined from the average of all three experiments (Table 1). *C*–*E*, the zinc-binding site from (*C*) the crystal structure of CspZ_B31_ (*blue*) and human SCR6-7 (*gray*) complex is compared with the equivalent site from (*D*) the crystal structure from CspZ_B408_ (*gold*) and human SCR6-7 complex (*lilac*, Protein Data Bank ID: 7ZJM), and (*E*) the human SCR6-7 (*gray*) modeled with predicted structure of CspZ_VS461_ (*green*). SPR, surface plasmon resonance.

We next sought to test the role of zinc in modulating the ability of CspZ to bind human FH using SPR. We chelated CspZ_B31_ by treating this protein with EDTA to chelate divalent metal ions. The untreated or EDTA-treated CspZ_B31_ were then allowed to interact with human FH to obtain the FH-binding affinity. Compared with untreated CspZ_B31_ (Fig. 1*B* and Table 1), we found an 8.9-fold increase in *K*_D_ values of EDTA-treated CspZ_B31_ (3.05 μM, *p* = 0.009; Fig. 4*A* and Table 1), suggesting a reduction in FH-binding affinity after the removal of metal ions. We then dialyzed the EDTA-treated CspZ_B31_, incubated with zinc chloride, and reassessed the binding affinity with SPR. The resulting *K*_D_ value was indistinguishable from that value from untreated CspZ_B31_ (0.68 μM, *p* = 0.08; Figs. 1*B* and 4*B*, and Table 1), showing the ability of zinc in facilitating CspZ_B31_-mediated human FH–binding activity. We treated the other human FH–binding CspZ variants (CspZ_B408_ and CspZ_V461_) with EDTA and determined their human FH–binding affinity in the same fashion. We found no significant difference of the *K*_D_ values between untreated and EDTA-treated CspZ_B408_ or CspZ_V461_ (*p* = 0.64 and 0.85 for CspZ_B408_ or CspZ_V461_, respectively; Figs. 1*B* and 4*A*, and Table 1). These results demonstrate that the zinc-coordinated FH-binding mode is CspZ variant specific.

To identify the molecular basis of the variant-specific and zinc-coordinated FH binding, we compared the zinc-binding pocket of the newly resolved structure of CspZ_B31_–SCR6-7 with the equivalent loci in the previously obtained CspZ_B408_–SCR6-7 complex structure (27). AlphaFold2 predicted structure of CspZ_VS461_ with SCR6-7 was also included. Similar to the zinc in the CspZ_B31_–SCR6-7 complex, zinc coordinated the binding of His402 of SCR7 and Glu65 of CspZ_B408_ in the CspZ_B408_–SCR6-7 complex (Fig. 4, *C* and *D*). However, because of the extension by three residues (Asn67–Asn68–Val69), the elongated loop B/C of CspZ_B408_ (Fig. S4) is altering the positioning of Glu65 in CspZ_B408_. This positional shift results in only one oxygen atom from the carboxyl group of Glu65 in CspZ_B408_ being involved in zinc coordination (Fig. 4*D*). It should be noted that a zinc ion was present in the precipitant solution used for crystallization of CspZ_B408_–SCR6-7 complex. Furthermore, CspZ_B408_ was previously found to bind only a single SCR6-7 molecule (Fig. 4*D*) (27). As the zinc-binding pocket in CspZ_B31_–SCR6-7 utilized two SCR6-7 molecules (Fig. 4*C*), the lack of binding a second SCR6-7 to CspZ_B408_ prevented the formation of a zinc-coordinated pocket made by three histidine residues and one glutamate residue. Overall, these structural differences between CspZ_B31_ and CspZ_B408_ in the loop B/C and adjacent regions provide evidence underlying the dispensable role of zinc in facilitating FH-binding activity of CspZ_B408_. Furthermore, negatively charged residues were not found at the equivalent location of CspZ_VS461_, and the glutamate residue located four amino acids upstream (Fig. S4) is too distant to interact with the zinc pocket. This finding addresses the metal-independent FH binding of CspZ_VS461_ (Fig. 4*E*). Taken together, these results suggest that the loop B/C conformation dictates Glu65 orientation, driving the allelic-specific role of zinc to promote CspZ-mediated human FH–binding activity.

### The role of Glu65-mediated FH binding correlates with the diversification of CspZ

**Figure 5:**
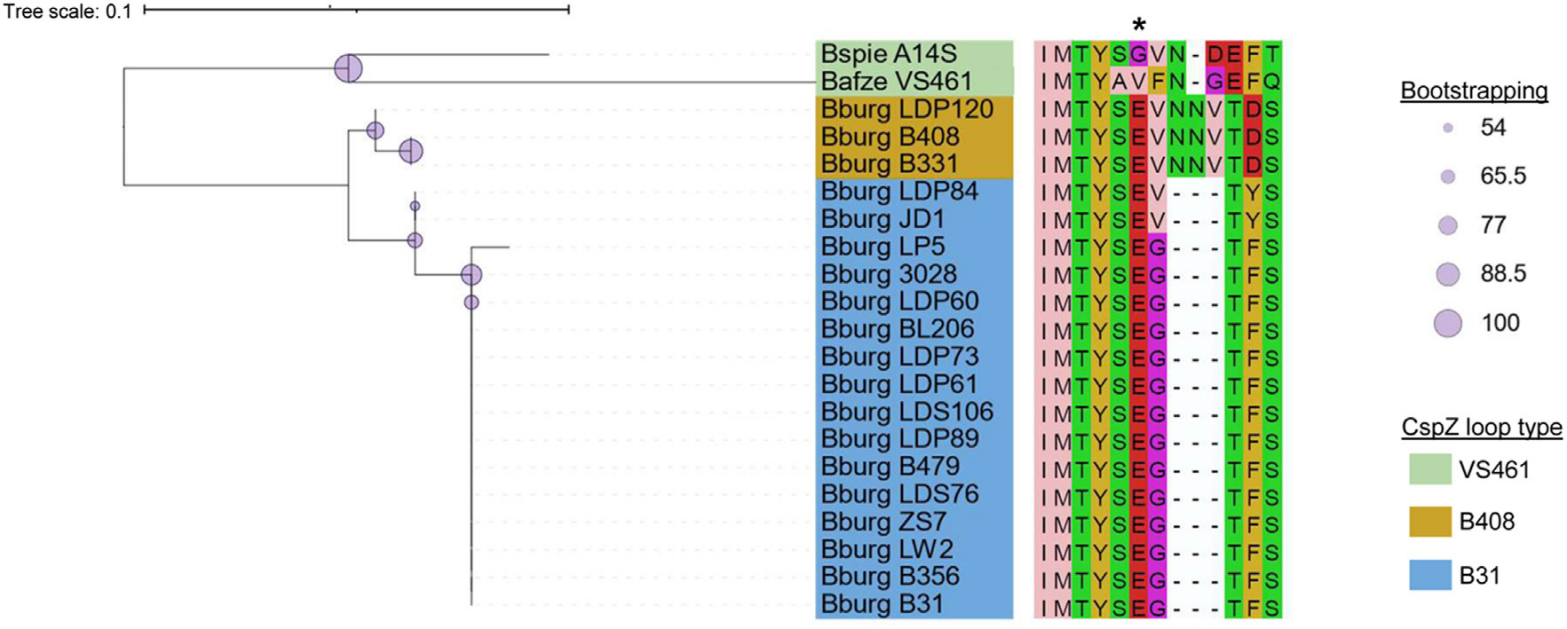
**Sequence comparisons support the Glu65 loop B/C topology correlates with the diversification of CspZ.** An unrooted maximum likelihood phylogenetic tree of human factor H-binding CspZ variants was generated with IQ-tree and visualized in iTOL. Bootstrapping values are indicated with the *lilac circles*, in which a *larger circle* correlates with a stronger bootstrapping value. The names of the isolates are highlighted based on their loop B/C types, relative to CspZ_B31_ (*blue*), CspZ_B408_ (*gold*), or CspZ_VS461_ (*green*). The protein alignment, highlighting the loop B/C region, shown to the *right*, was visualized with Jalview and colored with the Zappo scheme based on physiochemical properties. The *asterisk* indicates the Glu65 site in CspZ_B31_ or equivalent sites in other variants.

We previously observed three polymorphic CspZ variants within the *B. burgdorferi* sensu stricto population, correlating with distinct human FH–binding abilities (27). Herein, we noted that CspZ from two *B. afzelii* strains had differential human FH–binding abilities, and these CspZ variants were polymorphic (Fig. S4). These data suggest that CspZ variants within not only *B. burgdorferi* but other Lyme borreliae species may have evolved unique and variant-specific mechanisms of human FH binding. We thus curated the available CspZ sequences of all known human FH binders (28, 33, 34, 35, 36, 37, 38, 39) (Table S1) to generate a maximum-likelihood phylogenetic tree (Fig. 5). Although acknowledging the caveat that this dataset was primarily dominated by *B. burgdorferi* sensu stricto, the phylogenetic topology was still correlated with known species-level relationships (40): *B. afzelii* and *Borrelia spielmanii* CspZ variants were closely related in the same branch, whereas *B. burgdorferi* CspZ variants formed a separate lineage. In addition, *B. burgdorferi* CspZ variants were divided into two distinct branches, confirming previously determined relationships (27). When overlaying the alignment of the residues composing the CspZ loop B/C with this multispecies tree, we observed three distinct groups aligning with the variants studied herein: (1) CspZ_B31_-like, where variants harbored Glu65 and no insertion that altered its orientation; (2) CspZ_B408_-like, which harbored Glu65 and the insertion that altered its orientation; and (3) CspZ_VS461_-like, where there was no negatively charged residue at the Glu65 equivalent site (Fig. 5). These similarities observed in the loop B/C region correlate with the overall phylogenetic topography, despite a lack of clonality across the entire CspZ protein among variants within the same group. These findings suggest that across the wider human FH–binding Lyme borreliae population, CspZ appears to have evolved unique, variant-specific mechanisms for human FH binding, as demonstrated by the three variants extensively studied herein.

## Discussion

Metal ions are involved in many crucial biological processes during host–pathogen interactions (4). As metal ions often present in hosts at trace concentrations, pathogens are documented to develop strategies (*e.g.*, metal-binding transporters or acquiring proteins) in acquiring such crucial limited resources (41). Some pathogens have evolved strategies to use these metal ions to coordinate the pathogen protein—ligand interactions (42). In Lyme borreliae, the role of metal ions is largely unclear. Some work demonstrates the lack of iron-containing proteins and genes encoding iron-acquiring proteins from bacterial lysates, suggesting that they simply do not utilize this metal during infection (43). However, iron-, zinc-, cooper-, and/or manganese-binding proteins, and the transporter of some of these ions, have recently been identified in Lyme borreliae (19, 44). These ions can regulate the functions of metal transporters, metalloenzymes, or transcriptional factors (17, 45, 46, 47, 48). In addition, some of these metal ion–binding proteins promote pathogen evasion to reactive oxygen and nitrogen species, suggesting a role of metal ion(s) for Lyme borreliae to escape killing by innate immune system (18, 49). In this study, we observed that a zinc ion coordinates the binding of complement regulator FH to CspZ of *B. burgdorferi*, showing the requirement of this cation in facilitating CspZ-dependent human FH–binding activity. Such an activity was reported to confer complement immune evasion and pathogen dissemination in different vertebrate animal models (26, 27). Furthermore, Lyme disease infection progresses from acute and local symptoms associated with infection establishment (*i.e.*, erythema migrans) to late disseminated or chronic systemic infection manifestations (*e.g.*, arthritis, acrodermatitis chronica atrophicans, and neuroborreliosis) in humans (12). Therefore, our results may support the role of metal ions in facilitating the functions of Lyme borreliae immune evasion proteins to promote pathogen infectivity.

Lyme borreliae have diversified into multiple species and strains with distinct genotypes. The infection phenotypes of vertebrates, including humans, are linked to such genetic polymorphism (50). CspZ displays only minor sequence variations (∼2% and ∼20% in the variants amongst different strains and species, respectively), and this variation is linked to distinct FH-binding activity of different animals, including humans (27, 28, 36). The crystal structure of the CspZ_B31_–FH complex presented in this study identified a zinc-coordinated FH-binding site involving Glu65 in the CspZ loop B/C region, which is polymorphic among variants (27), and this binding incorporated two FH molecules. The other FH molecule participates in zinc coordination through two of its histidines, specifically SCR6 residues, His371 and His373 (Fig. 3*E*). In addition, the second FH molecule interacts with CspZ *via* the SCR6 domain using Ser367 and Asp370 (Fig. 3*E*). This observation suggests that two FH molecules could bind to the CspZ protein *in vivo* in a similar manner. Supporting this, when the full-length human FH structure predicted by AlphaFold is superimposing with both SCR6-7 molecules observed in the CspZ_B31_–SCR6-7 crystal structure, it becomes evident that binding of two FH molecules simultaneously is potentially feasible (Fig. S6). Furthermore, we found that the FH-binding variant CspZ_VS461_ lacks an equivalent amino acid to Glu65 at this position. In contrast, the other FH-binder, CspZ_B408_, does possess Glu65 on its loop B/C. However, because of the extension by several residues, the conformation of the loop region harboring Glu65 in CspZ_B408_ differs from that of CspZ_B31_, and no additional FH molecule was incorporated into the binding. These characteristics resulted in the dispensable role of zinc in promoting human FH binding to CspZ_B408_. Furthermore, CspZ-mediated FH-binding activity was reported to facilitate Lyme borreliae dissemination *in vivo*. Our findings thus would provide a mechanism underlying the documented correlation of Lyme borreliae genetic variation with their distinct dissemination potential in patients (13, 14, 15).

Our results also support that there are multiple modes of FH binding for CspZ variants. In addition to Glu65, which coordinates zinc-mediated binding of CspZ_B31_ to human FH, CspZ_B31_, CspZ_B408,_ and CspZ_VS461_ vary in their amino acids involved in FH binding. For example, Asp71 in CspZ_B408_ is involved in human FH binding by mediating electrostatic interactions with Lys388 and Tyr390 from human SCR6-7, while the equivalent amino acid is absent in CspZ_B31_ (Fig. S4) (27). Despite these notable differences in the FH-binding interface of CspZ_B31_, CspZ_B408,_ and CspZ_VS461_, we did not see significant differences of the human FH–binding *K*_D_ values for these three proteins. This raises the intriguing possibility of compensatory mutations, where the loss of fitness caused by one mutation within a protein is remedied by secondary mutations at other sites (51), and the emergence of such compensatory mutations can prevent eradication because of selective pressure (52). In our case, the zinc-mediated human FH binding observed in CspZ_B31_ may serve as a compensatory mechanism for the loss of the Asp71 residue seen in CspZ_B408_ or *vice versa*. The complete lack of Glu65 in CspZ_VS461_ suggests another binding mechanism evolved, which may be compensatory for the loss of Glu65. Albeit limited because very few CspZ variants have been evaluated for their ability to bind human FH, our phylogenetic analysis illustrated distinct evolutionary trajectories among the lineages containing these three variants, supporting the possibility of unique compensatory mutations within the CspZ proteins. To further explore this model, additional work involving large-scale sequencing, evolutionary ancestral predictions, and functional testing will be necessary.

In this study, we used the Lyme borreliae protein CspZ and its target ligand FH as a model to show the role of a metal ion in promoting the binding of a pathogen protein to a key complement regulator of the alternative pathway. We identified the variant-specific requirement for zinc in promoting this binding activity to immune inhibitory proteins, providing a potential mechanistic understanding that links genetic polymorphism with pathogen immune evasion phenotypes. In addition, comparing the structural basis of zinc-promoted infectious phenotypes with the phylogeny of the pathogen protein supports the notion that polymorphisms drive the evolution of immune evasion proteins. By investigating the role of metal ions in enabling pathogen proteins to perform their functions, this work ultimately would pave the way for the development of new treatment strategies against microbial pathogen–associated diseases.

## Experimental procedures

### Ethics statement

Collection of blood samples and consent documents was approved by the Ethics Committee at the University Hospital of Frankfurt (control number 160/10 and 222/14), Goethe University of Frankfurt am Main. All healthy blood donors provided written informed consent in accordance with the Declaration of Helsinki.

### Bacterial strains, sera, antibodies, and proteins

The *Borrelia* and *Escherichia coli* strains used in this study are described in Table S2. *E. coli* strains and derivatives were grown in Luria–Bertani (BD Bioscience) broth or agar, supplemented with ampicillin (100 μg/ml) or no antibiotics as appropriate. All *Borrelia* strains were grown in Barbour–Stoenner–Kelly-H (BSK-H) medium (Bio&SELL). *B. garinii* transformants were growth in BSK-H supplemented with streptomycin (100 μg/ml). Nonimmune human serum (NHS) collected from healthy blood donors was initially tested for the presence of anti-*Borrelia* IgM and IgG antibodies as previously described (53, 54). Only sera considered to be negative were combined to form a serum pool. Polyclonal anti-FH and anti-C3 antisera were obtained from Merck Biosciences, and the neoepitope-specific monoclonal anti-C5b-9 antibody was purchased from Quidel. Serum-purified complement proteins (C3b, FI, and FH) were obtained from Complement Technology. BSA, trypsin, and proteinase K were purchased from Merck. Polyclonal antisera raised against complement C3 and FH were from Merck. The polyclonal anti-GST antibody was from GE Healthcare. Horseradish peroxidase–conjugated immunoglobulins were obtained from Agilent Technologies Denmark, and Alexa Fluor 488-conjugated immunoglobulins were from Invitrogen. Anti-*Borrelia* monoclonal antibodies L41 1C11 against FlaB and anti-CspZ polyclonal antibodies were obtained as described previously (29, 55).

### Expression and purification of CspZ and human SCR6-7

The genes encoding different CspZ variants were amplified from the genomic DNA of *B. burgdorferi*, *B. afzelii*, or *B. garinii* strains (Table S2). The primers to amplify those sequences are described in Table S3. The coding sequences were then ligated into the respective expression vectors indicated in Table S2. Protein expression and the subsequent purification of histidine-tagged or GST-tagged proteins were described previously (27, 30). For the proteins needed in an untagged version, we performed 6xHis proteolytic digestion by tobacco etch virus protease and removal of the cleaved 6xHis fragments, as described (31). The production of human FH SCR 6-7 was as described previously (27).

### ELISA

Microtiter plates (Thermo Fisher Scientific) were coated with 5 μg/ml of GST-tagged proteins or BSA in PBS at 4 °C as described (53, 54). Wells were washed three times with PBS containing 0.05% (v/v) Tween-20 (PBS-T) and then blocked with Blocking Buffer III BSA (AppliChem). Following three washes with PBS-T, purified FH (5 μg/ml) was added. After incubation for 1 h at room temperature, the wells were washed thoroughly with PBS-T, and the binding of FH was then assessed by a polyclonal goat anti-FH antiserum (1:1000 dilution). After washing, the protein complexes were detected by using horseradish peroxidase–conjugated antigoat immunoglobulins (1:2000 diltuion). O-phenylenediamine (Merck) and H_2_O_2_ were added to the wells, and the absorbance was measured at 490 nm.

### SPR analyses

Recombinant CspZ proteins (20 μM) were mixed with PBS or 100 μM of EDTA, followed by dialysis in 4 l of PBS buffer. Some of the EDTA-treated PBS-dialyzed recombinant CspZ_B31_ and CspZ_B408_ (20 μM) was further incubated with 100 μM of zinc chloride. The interactions of those CspZ proteins under different treatments with FH were analyzed by SPR using a Biacore T200 (Cytiva). About 10 μg of human FH were conjugated to a CM5 chip (Cytiva) as described previously (56). For quantitative SPR experiments, 10 μl of increasing concentrations (0.08, 0.03125, 0.0125, 0.5, and 2 μM) of untreated, EDTA-, or zinc chloride–treated CspZ_B31_ and CspZ_B408_ were injected into the control cell and the flow cell immobilized with FH at 10 μl/min of PBS at 25^o^C. The PBS buffer used is either untreated (for untreated CspZ), EDTA treated (100 μM; for EDTA-treated CspZ), or zinc chloride–treated PBS (100 μM; for zinc chloride–treated CspZ). To obtain the kinetic parameters of the interaction, sonogram data were fitted by means of BIAevaluation software version 3.0 (GE Healthcare), using the one-step biomolecular association reaction model (1:1 Langmuir model), resulting in optimum mathematical fit with the lowest Chi-square values.

Note that the kinetic parameters derived from lower flow rates of SPR experiments may not be accurate because the low flow rates of the molecules flowing through the SPR chip may result in the effects of mass transfer. Such mass transfer can cause the reduced *k*_on_ and *K*_D_ values for the interaction at low flow rate, compared with those at high flow rates. Thus, we determined all CspZ–FH interactions that showed detectable binding under flow rates of 10, 30, and 100 μl/min. In this flow-rate experiment, 2 μM of the indicated CspZ variants under the aforementioned treatment of EDTA and/or zinc chloride and then flow through the chips conjugated with FH. The kinetic parameters were obtained as described previously. Indistinguishable *k*_on_ and *K*_D_ values were observed in the interaction under the same treatment (Fig. S7 and Table S4), suggesting that the flow rates for the rest SPR experiments at 10 μl/min could provide accurate kinetic parameters.

### Western blotting

Reaction mixtures from the C3b cofactor and serum adsorption assays were separated by 10% Tris–tricine SDS-PAGE and transferred to nitrocellulose membranes as described previously (53). Briefly, the membranes were blocked with 5% nonfat dry milk in Tris-buffered saline (TBS) containing 0.1% Tween-20. After three wash steps with TBS containing 0.1% Tween-20, membranes were incubated with an anti-CspZ, anti-human C3b, or anti-human FH antibody, followed by horseradish peroxidase–conjugated antimouse immunoglobulins (1:1000 dilution) (53). Protein–antigen complexes were detected by tetramethylbenzidine as substrate as described (53). Images of the gels and nitrocellulose membranes were processed by using a GS-710 image densitometer (Bio-Rad) and the Quantity One software version 4.2.1 (Bio-Rad).

### Serum adsorption assay

To detect binding of serum-derived FH to the surface of transformed bacteria, spirochetes grown at midlogarithmic phase were sedimented. Then, 2 × 10^9^ of the spirochetes were incubated in 750 μl NHS–EDTA for 1 h at room temperature as described previously (29). After washing the spirochetes with PBS, the last wash fraction was collected, and surface-bound proteins were then eluted by using 0.1 M glycine–HCl, pH 2.0 and analyzed by Western blotting as previously described (29).

### C3b cofactor assay

FI-mediated C3b inactivation was assayed after immobilization of purified GST-tagged proteins (100 ng/ml) or PBS-washed spirochetes (2 × 10^8^ cells in 100 μl) on microtiter plates at 4 °C as described previously (57). Briefly, the wells were incubated with either 1 μg FH (purified proteins) or 0.5 μg FH (spirochetes) for 1 h at room temperature. After washing, PBS containing C3b (10 μg/ml) and FI (20 μg/ml) was added. Following incubation, SDS-PAGE sample buffer was added, and each reaction mixture was then loaded on a 10% Tris–tricine SDS polyacrylamide gel. After transfer to nitrocellulose membranes, C3b cleavage products were analyzed by Western blotting using a polyclonal anti-C3 antibody.

### Immunofluorescence microscopy

Deposition of activated complement components was visualized by immunofluorescence microscopy as previously described (53). Spirochetes (6 × 10^6^) were incubated with 25% NHS for 30 min at 37 °C with gentle agitation, washed three times with PBS containing 1% BSA. Aliquots of 12 μl were then spotted on microscope slides and allowed to air dry overnight. After fixation with glyoxal, slides were incubated for 1 h in a humidified chamber with antibodies against complement components C3 (dilution of 1:1000) and C5b-9 (dilution of 1:70). Following four washes with PBS, the slides were incubated for 1 h at room temperature with 1:2000 dilutions of Alexa 488-conjugated secondary antibodies (Life Technologies). Slides were then washed, sealed, and visualized by using an Axio Imager M2 fluorescence microscope (Zeiss) equipped with a Spot RT3 camera (Visitron Systems).

### Serum resistance assay

Spirochetes were grown to midlogarithmic phase, viability was confirmed visually, and sedimented by centrifugation, and resuspended in 500 μl BSK-H medium. Reaction mixtures consisting of 50 μl spirochetes (1 × 10^7^) and 50 μl of 50% NHS were incubated at 37 °C with gentle agitation. The percentage of motile and viable cells was determined by dark field microscopy after 0, 1, 2, 4, and 6 h, respectively. Nine microscopy fields were counted for each time point per analyzed strain, and each test was performed at least three times as previously described (29, 53, 57).

### The confirmation of surface localization by protease degradation assays

Localization of CspZ-producing spirochetes was assessed by an *in situ* protease degradation assay as previously described (58). Briefly, borrelial cells (1.4 × 10^9^) were incubated with increasing concentrations of proteinase K or trypsin (12.5–50 μg/ml) for 2 h at room temperature. All reactions were terminated by adding 5 μl phenylmethylsulfonyl fluoride (Merck) and 71 μl of complete protease inhibitor cocktail (Merck). After centrifugation, spirochetes were washed twice and lysed by sonication. The lysates were subjected to 10% Tris–tricine SDS-PAGE and then Western blotting as described previously.

### Crystallization, data collection, and structure determination

Prior to crystallization, equal volumes of 3.6 mg/ml CspZ and 3.5 mg/ml SCR6-7 were mixed together and loaded onto a HiLoad 16/600 Superdex 200 prep grade gel-filtration column (GE Healthcare). The fractions corresponding to the main peak were pulled and concentrated to 10 mg/ml using an Amicon centrifugal filter unit (Millipore) and analyzed by SDS-PAGE to confirm the formation of the CspZ_B31_–SCR6-7 complex. Crystallization was performed using a Tecan Freedom EVO100 workstation (Tecan Group) by mixing 0.4 μl of CspZ_B31_–SCR6-7 protein complex with 0.4 μl of precipitant. The 96-reagent sparse-matrix screens JCSG+ and Structure Screen 1 & 2 (Molecular Dimensions) were used as precipitant. The crystals used for diffraction analysis and X-ray absorption spectroscopy were obtained in a precipitant solution containing 0.25 M ammonium sulfate, 0.1 M Mes (pH 6.5), and 10% PEG 3000. The corresponding precipitant solution containing 10% glycerol was used as a cryoprotectant prior to transfer in liquid nitrogen. The diffraction data and X-ray absorption spectroscopy data were collected at the MX beamline instrument BL14.1 at the BESSY II electron storage ring operated by the Helmholtz-Zentrum, Berlin (32). Reflections were indexed by XDS and scaled by AIMLESS from the CCP4 suite (59, 60, 61). CspZ_B31_–SCR6-7 was solved by molecular replacement using *B. burgdorferi* CspZ_B408_ and human FH SCR domains 6-7 complex structure as a search model (Protein Data Bank ID: 7ZJM). BUCCANEER (62) was used to automatically built the protein chain into the electron density followed by manual rebuilding in COOT (63). Crystallographic refinement was performed using REFMAC5 (64). A summary of the data collection, refinement, and validation statistics for CspZ_B31_–SCR6-7 complex is given in Table S4.

### Protein 3D structure prediction using AlphaFold2

AlphaFold, v2.0 (65) was used to predict the 3D structure for CspZ_VS461_ (GenBank ID: QHO60340.1) and full-length human FH (UniProt entry: P08603). Structure prediction with AlphaFold v2.0 was performed according to the default parameters as described previously for PFam12 member proteins (66).

### Alignment and phylogenetic analyses of CspZ variants

We pulled CspZ from *B. burgdorferi* B31 (GenBank ID: AAC65998.1), B408 (GenBank ID: UNE56005.1), *B. afzelii* VS461 (GeneBank ID: QHO60340.1), and *B. afzelii* FEM4 (GeneBank ID: OM243915.1). We have previously analyzed CspZ from two clones of VS461: VS461-JL (accession: QHO60339.1) and VS461-PK (accession: QHO60340.1). The former was used exclusively herein, but these two clones have identical CspZ sequences (37). The amino acid sequences were aligned in MEGA 11 (67) with clustalW, and on the tcoffee (68) and mcoffee (69) webservers, all at default settings. The alignment scores were calculated with TCS (70) at defaults, and the highest scoring alignment was visualized with Jalview v2.11.3.3 (71). These methods were repeated for the alignment of all CspZ variants that bind to human FH, with the exception that the signal peptide was detected and removed (SignalP v6.0) (72) because many variants did not have the signal peptide included in the available sequence. The alignment of all CspZ sequences was used to generate a phylogenetic tree with the IQ-TREE webserver (73) as previously described (27), which was visualized with iTOL v6.9.1 (74).

### Statistical analysis

Samples were compared using one-way ANOVA with Bonferroni’s multiple comparison post hoc test (95% confidence interval), which was conducted by applying GraphPad Prism, version 7 (GraphPad Software, Inc) (75).

### Accession numbers

The coordinates and the structure factors for CspZ_B31_–SCR6-7 complex have been deposited in the Protein Data Bank with accession code 9F7I.

## Data availability

All data are contained within this article.

## Supporting information

This article contains supporting information (21, 26, 27, 28, 30, 31, 33, 34, 35, 37, 38, 39, 50, 76, 77, 78, 79, 80).

## Conflict of interest

The authors declare that they have no conflicts of interest with the contents of this article.

## References

[1] Sheppard S.K., Guttman D.S., Fitzgerald J.R. (2018). Population genomics of bacterial host adaptation. Nat. Rev. Genet..

[2] Gatt Y.E., Margalit H. (2021). Common adaptive strategies underlie within-host evolution of bacterial pathogens. Mol. Biol. Evol..

[3] Didelot X., Walker A.S., Peto T.E., Crook D.W., Wilson D.J. (2016). Within-host evolution of bacterial pathogens. Nat. Rev. Microbiol..

[4] Begg S.L. (2019). The role of metal ions in the virulence and viability of bacterial pathogens. Biochem. Soc. Trans..

[5] Nunez G., Sakamoto K., Soares M.P. (2018). Innate nutritional immunity. J. Immunol..

[6] Porcheron G., Garenaux A., Proulx J., Sabri M., Dozois C.M. (2013). Iron, copper, zinc, and manganese transport and regulation in pathogenic Enterobacteria: correlations between strains, site of infection and the relative importance of the different metal transport systems for virulence. Front. Cell. Infect. Microbiol..

[7] Hagan E.C., Mobley H.L. (2009). Haem acquisition is facilitated by a novel receptor Hma and required by uropathogenic *Escherichia coli* for kidney infection. Mol. Microbiol..

[8] Garcia E.C., Brumbaugh A.R., Mobley H.L. (2011). Redundancy and specificity of *Escherichia coli* iron acquisition systems during urinary tract infection. Infect. Immun..

[9] Rossi M.S., Fetherston J.D., Letoffe S., Carniel E., Perry R.D., Ghigo J.M. (2001). Identification and characterization of the hemophore-dependent heme acquisition system of *Yersinia pestis*. Infect. Immun..

[10] Hu L.T. (2016). Lyme disease. Ann. Intern. Med..

[11] Steere A.C., Strle F., Wormser G.P., Hu L.T., Branda J.A., Hovius J.W. (2016). Lyme borreliosis. Nat. Rev. Dis. primers.

[12] Radolf J.D., Strle K., Lemieux J.E., Strle F. (2021). Lyme disease in humans. Curr. Issues Mol. Biol..

[13] Strle K., Jones K.L., Drouin E.E., Li X., Steere A.C. (2011). Borrelia burgdorferi RST1 (OspC type A) genotype is associated with greater inflammation and more severe Lyme disease. Am. J. Pathol..

[14] Cerar T., Strle F., Stupica D., Ruzic-Sabljic E., McHugh G., Steere A.C. (2016). Differences in genotype, clinical features, and inflammatory potential of *Borrelia burgdorferi* sensu stricto strains from europe and the United States. Emerging Infect. Dis..

[15] Lemieux J.E., Huang W., Hill N., Cerar T., Freimark L., Hernandez S. (2023). Whole genome sequencing of human *Borrelia burgdorferi* isolates reveals linked blocks of accessory genome elements located on plasmids and associated with human dissemination. PLoS Pathog..

[16] Troxell B., Yang X.F. (2013). Metal-dependent gene regulation in the causative agent of Lyme disease. Front. Cell. Infect. Microbiol..

[17] Troxell B., Ye M., Yang Y., Carrasco S.E., Lou Y., Yang X.F. (2013). Manganese and zinc regulate virulence determinants in *Borrelia burgdorferi*. Infect. Immun..

[18] Troxell B., Xu H., Yang X.F. (2012). *Borrelia burgdorferi*, a pathogen that lacks iron, encodes manganese-dependent superoxide dismutase essential for resistance to streptonigrin. J. Biol. Chem..

[19] Ouyang Z., He M., Oman T., Yang X.F., Norgard M.V. (2009). A manganese transporter, BB0219 (BmtA), is required for virulence by the Lyme disease spirochete, *Borrelia burgdorferi*. Proc. Natl. Acad. Sci. U. S. A..

[20] Skare J.T., Garcia B.L. (2020). Complement evasion by Lyme disease spirochetes. Trends Microbiol..

[21] Lin Y.P., Diuk-Wasser M.A., Stevenson B., Kraiczy P. (2020). Complement evasion contributes to Lyme borreliae-host associations. Trends Parasitology.

[22] Dulipati V., Meri S., Panelius J. (2020). Complement evasion strategies of *Borrelia burgdorferi* sensu lato. FEBS Lett..

[23] Reis E.S., Mastellos D.C., Hajishengallis G., Lambris J.D. (2019). New insights into the immune functions of complement. Nat. Rev. Immunol..

[24] Lin Y.P., Frye A.M., Nowak T.A., Kraiczy P. (2020). New insights into CRASP-mediated complement evasion in the Lyme disease enzootic cycle. Front. Cell. Infect. Microbiol..

[25] Bykowski T., Woodman M.E., Cooley A.E., Brissette C.A., Brade V., Wallich R. (2007). Coordinated expression of *Borrelia burgdorferi* complement regulator-acquiring surface proteins during the Lyme disease spirochete's mammal-tick infection cycle. Infect. Immun..

[26] Marcinkiewicz A.L., Dupuis A.P., Zamba-Campero M., Nowak N., Kraiczy P., Ram S. (2019). Blood treatment of Lyme borreliae demonstrates the mechanism of CspZ-mediated complement evasion to promote systemic infection in vertebrate hosts. Cell Microbiol..

[27] Marcinkiewicz A.L., Brangulis K., Dupuis A.P., Hart T.M., Zamba-Campero M., Nowak T.A. (2023). Structural evolution of an immune evasion determinant shapes pathogen host tropism. Proc. Natl. Acad. Sci. U. S. A..

[28] Rogers E.A., Abdunnur S.V., McDowell J.V., Marconi R.T. (2009). Comparative analysis of the properties and ligand binding characteristics of CspZ, a factor H binding protein, derived from *Borrelia burgdorferi* isolates of human origin. Infect. Immun..

[29] Siegel C., Schreiber J., Haupt K., Skerka C., Brade V., Simon M.M. (2008). Deciphering the ligand-binding sites in the *Borrelia burgdorferi* complement regulator-acquiring surface protein 2 required for interactions with the human immune regulators factor H and factor H-like protein 1. J. Biol. Chem..

[30] Nowak T.A., Lown L.A., Marcinkiewicz A.L., Surth V., Kraiczy P., Burke R. (2023). Outer surface protein E (OspE) mediates *Borrelia burgdorferi* sensu stricto strain-specific complement evasion in the eastern fence lizard, Sceloporus undulatus. Ticks tick-borne Dis..

[31] Brangulis K., Petrovskis I., Kazaks A., Bogans J., Otikovs M., Jaudzems K. (2014). Structural characterization of CspZ, a complement regulator factor H and FHL-1 binding protein from *Borrelia burgdorferi*. FEBS J..

[32] Mueller U., Darowski N., Fuchs M.R., Forster R., Hellmig M., Paithankar K.S. (2012). Facilities for macromolecular crystallography at the helmholtz-zentrum Berlin. J. Synchrotron Radiat..

[33] Marcinkiewicz A.L., Lieknina I., Kotelovica S., Yang X., Kraiczy P., Pal U. (2018). Eliminating factor H-binding activity of *Borrelia burgdorferi* CspZ combined with virus-like particle conjugation enhances its efficacy as a Lyme disease vaccine. Front. Immunol..

[34] Muhleip J.J., Lin Y.P., Kraiczy P. (2018). Further insights into the interaction of human and animal complement regulator factor H with viable Lyme disease spirochetes. Front. Vet. Sci..

[35] Kraiczy P., Skerka C., Brade V., Zipfel P.F. (2001). Further characterization of complement regulator-acquiring surface proteins of *Borrelia burgdorfer*i. Infect. Immun..

[36] Rogers E.A., Marconi R.T. (2007). Delineation of species-specific binding properties of the CspZ protein (BBH06) of Lyme disease spirochetes: evidence for new contributions to the pathogenesis of *Borrelia* spp. Infect. Immun..

[37] Marcinkiewicz A.L., Lieknina I., Yang X., Lederman P.L., Hart T.M., Yates J. (2020). The factor H-binding site of CspZ as a protective target against multistrain, tick-transmitted Lyme disease. Infect. Immun..

[38] Kraiczy P., Skerka C., Kirschfink M., Brade V., Zipfel P.F. (2001). Immune evasion of *Borrelia burgdorferi* by acquisition of human complement regulators FHL-1/reconectin and Factor H. Eur. J. Immunol..

[39] Herzberger P., Siegel C., Skerka C., Fingerle V., Schulte-Spechtel U., van Dam A. (2007). Human pathogenic *Borrelia spielmanii* sp. nov. resists complement-mediated killing by direct binding of immune regulators factor H and factor H-like protein 1. Infect. Immun..

[40] Schwartz I., Margos G., Casjens S.R., Qiu W.G., Eggers C.H. (2021). Multipartite genome of Lyme disease *Borrelia*: structure, variation and prophages. Curr. Issues Mol. Biol..

[41] Lopez C.A., Skaar E.P. (2018). The impact of dietary transition metals on host-bacterial interactions. Cell Host & Microbe..

[42] German N., Luthje F., Hao X., Ronn R., Rensing C. (2016). Microbial virulence and interactions with metals. Prog. Mol. Biol. Translational Sci..

[43] Posey J.E., Gherardini F.C. (2000). Lack of a role for iron in the Lyme disease pathogen. Science.

[44] Wang P., Lutton A., Olesik J., Vali H., Li X. (2012). A novel iron- and copper-binding protein in the Lyme disease spirochaete. Mol. Microbiol..

[45] Nguyen K.T., Wu J.C., Boylan J.A., Gherardini F.C., Pei D. (2007). Zinc is the metal cofactor of *Borrelia burgdorferi* peptide deformylase. Arch. Biochem. Biophys..

[46] Russell T.M., Tang X., Goldstein J.M., Bagarozzi D., Johnson B.J. (2016). The salt-sensitive structure and zinc inhibition of *Borrelia burgdorferi* protease BbHtrA. Mol. Microbiol..

[47] Wang P., Yu Z., Santangelo T.J., Olesik J., Wang Y., Heldwein E. (2017). BosR is A novel Fur family member responsive to copper and regulating copper homeostasis in *Borrelia burgdorferi*. J. Bacteriol..

[48] Wagh D., Pothineni V.R., Inayathullah M., Liu S., Kim K.M., Rajadas J. (2015). Borreliacidal activity of *Borrelia* metal transporter A (BmtA) binding small molecules by manganese transport inhibition. Drug Des. Devel Ther..

[49] Aguirre J.D., Clark H.M., McIlvin M., Vazquez C., Palmere S.L., Grab D.J. (2013). A manganese-rich environment supports superoxide dismutase activity in a Lyme disease pathogen, *Borrelia burgdorferi*. J. Biol. Chem..

[50] Tufts D.M., Hart T.M., Chen G.F., Kolokotronis S.O., Diuk-Wasser M.A., Lin Y.P. (2019). Outer surface protein polymorphisms linked to host-spirochete association in Lyme borreliae. Mol. Microbiol..

[51] Kimura M. (1985). The role of compensatory neutral mutations in molecular evolution. J. Genet..

[52] Camps M., Herman A., Loh E., Loeb L.A. (2007). Genetic constraints on protein evolution. Crit. Rev. Biochem. Mol. Biol..

[53] Hammerschmidt C., Koenigs A., Siegel C., Hallstrom T., Skerka C., Wallich R. (2014). Versatile roles of CspA orthologs in complement inactivation of serum-resistant Lyme disease spirochetes. Infect. Immun..

[54] Hammerschmidt C., Koenigs A., Siegel C., Hallstrom T., Skerka C., Wallich R. (2014). Versatile roles of CspA orthologs in complement inactivation of serum-resistant Lyme disease spirochetes. Infect. Immun..

[55] Hauser U., Lehnert G., Wilske B. (1999). Validity of interpretation criteria for standardized Western blots (immunoblots) for serodiagnosis of Lyme borreliosis based on sera collected throughout Europe. J. Clin. Microbiol..

[56] Lin Y.P., Greenwood A., Nicholson L.K., Sharma Y., McDonough S.P., Chang Y.F. (2009). Fibronectin binds to and induces conformational change in a disordered region of leptospiral immunoglobulin-like protein B. J. Biol. Chem..

[57] Walter L., Surth V., Rottgerding F., Zipfel P.F., Fritz-Wolf K., Kraiczy P. (2019). Elucidating the immune evasion mechanisms of *Borrelia mayonii*, the causative agent of Lyme disease. Front. Immunol..

[58] Hartmann K., Corvey C., Skerka C., Kirschfink M., Karas M., Brade V. (2006). Functional characterization of BbCRASP-2, a distinct outer membrane protein of *Borrelia burgdorferi* that binds host complement regulators factor H and FHL-1. Mol. Microbiol..

[59] Winn M.D., Ballard C.C., Cowtan K.D., Dodson E.J., Emsley P., Evans P.R. (2011). Overview of the CCP4 suite and current developments. Acta Crystallographica. Section D, Biol. Crystallogr..

[60] Evans P.R. (2011). An introduction to data reduction: space-group determination, scaling and intensity statistics. Acta Crystallogr. D Biol. Crystallogr..

[61] Kabsch W. (2010). Xds. Acta Crystallogr. D Biol. Crystallogr..

[62] Cowtan K. (2006). The Buccaneer software for automated model building. 1. Tracing protein chains. Acta Crystallogr. D Biol. Crystallogr..

[63] Emsley P., Cowtan K. (2004). Coot: model-building tools for molecular graphics. Acta Crystallogr. D Biol. Crystallogr..

[64] Murshudov G.N., Vagin A.A., Dodson E.J. (1997). Refinement of macromolecular structures by the maximum-likelihood method. Acta Crystallogr. D Biol. Crystallogr..

[65] Jumper J., Evans R., Pritzel A., Green T., Figurnov M., Ronneberger O. (2021). Highly accurate protein structure prediction with AlphaFold. Nature.

[66] Brangulis K., Akopjana I., Drunka L., Matisone S., Zelencova-Gopejenko D., Bhattacharya S. (2024). Members of the paralogous gene family 12 from the Lyme disease agent *Borrelia burgdorferi* are non-specific DNA-binding proteins. PLoS One.

[67] Tamura K., Stecher G., Kumar S. (2021). MEGA11: molecular evolutionary genetics analysis version 11. Mol. Biol. Evol..

[68] Di Tommaso P., Moretti S., Xenarios I., Orobitg M., Montanyola A., Chang J.M. (2011). T-Coffee: a web server for the multiple sequence alignment of protein and RNA sequences using structural information and homology extension. Nucleic Acids Res..

[69] Moretti S., Armougom F., Wallace I.M., Higgins D.G., Jongeneel C.V., Notredame C. (2007). The M-Coffee web server: a meta-method for computing multiple sequence alignments by combining alternative alignment methods. Nucleic Acids Res..

[70] Chang J.M., Di Tommaso P., Notredame C. (2014). TCS: a new multiple sequence alignment reliability measure to estimate alignment accuracy and improve phylogenetic tree reconstruction. Mol. Biol. Evol..

[71] Waterhouse A.M., Procter J.B., Martin D.M., Clamp M., Barton G.J. (2009). Jalview Version 2--a multiple sequence alignment editor and analysis workbench. Bioinformatics.

[72] Teufel F., Almagro Armenteros J.J., Johansen A.R., Gislason M.H., Pihl S.I., Tsirigos K.D. (2022). SignalP 6.0 predicts all five types of signal peptides using protein language models. Nat. Biotechnol..

[73] Trifinopoulos J., Nguyen L.T., von Haeseler A., Minh B.Q. (2016). W-IQ-TREE: a fast online phylogenetic tool for maximum likelihood analysis. Nucleic Acids Res..

[74] Letunic I., Bork P. (2024). Interactive Tree of Life (iTOL) v6: recent updates to the phylogenetic tree display and annotation tool. Nucleic Acids Res..

[75] Bonferroni C.E. (1936).

[76] Purser J.E., Lawrenz M.B., Caimano M.J., Howell J.K., Radolf J.D., Norris S.J. (2003). A plasmid-encoded nicotinamidase (PncA) is essential for infectivity of *Borrelia burgdorferi* in a mammalian host. Mol. Microbiol..

[77] Baranton G., Postic D., Saint Girons I., Boerlin P., Piffaretti J.C., Assous M. (1992). Delineation of *Borrelia burgdorferi* sensu stricto, *Borrelia garinii* sp. nov., and group VS461 associated with Lyme borreliosis. Int. J. Syst. Bacteriol..

[78] Wilske B., Preac-Mursic V., Gobel U.B., Graf B., Jauris S., Soutschek E. (1993). An OspA serotyping system for *Borrelia burgdorferi* based on reactivity with monoclonal antibodies and OspA sequence analysis. J. Clin. Microbiol..

[79] Kraiczy P., Hunfeld K.P., Breitner-Ruddock S., Wurzner R., Acker G., Brade V. (2000). Comparison of two laboratory methods for the determination of serum resistance in *Borrelia burgdorferi* isolates. Immunobiology.

[80] Kraiczy P., Seling A., Brissette C.A., Rossmann E., Hunfeld K.P., Bykowski T. (2008). *Borrelia burgdorferi* complement regulator-acquiring surface protein 2 (CspZ) as a serological marker of human Lyme disease. Clin. Vaccin. Immunol..

