# Supplementary material for: CspZ variant–specific interaction with factor H incorporates a metal site to support Lyme borreliae complement evasion
Source: J Biol Chem. 2024 Dec 14;301(1):108083. doi: 10.1016/j.jbc.2024.108083 (PMC11773018; doi:10.1016/j.jbc.2024.108083)
Supplement: Supporting information [file mmc1.pdf]

Supporting information for

CspZ variant-specific interaction with Factor H incorporates a metal site to support Lyme borreliae complement evasion

Kalvis Brangulis, Valerie Sürth, Ashley L. Marcinkiewicz, Inara Akopjana, Andris Kazaks,

Janis Bogans, Alisa Huber, Yi-Pin Lin, Peter Kraiczy

Correspondence: Kalvis Brangulis, Ph.D.
Latvian Biomedical Research and Study Centre,
Ratsupites 1 k-1, LV-1067, Riga LV-1067, Latvia
Telephone: +371 6780 8200
Email: kalvis@biomed.lu.lv

Correspondence: Yi-Pin Lin, Ph.D.
Department of Infectious Disease and Global health,
Cummings School of Veterinary Medicine, Tufts University
North Grafton, MA USA
Telephone: +1 508-887-4535
Email: Yi-Pin.Lin@tufts.edu

Correspondence: Peter Kraiczy, Ph.D.
Institute of Medical Microbiology and Infection Control,
University Hospital, Goethe University Frankfurt,
Paul-Ehrlich-Str. 40, Frankfurt am Main, D60596, Germany
Telephone: +49-69-6301-7165
Email: Kraiczy@em.uni-frankfurt.de

This PDF file includes:
Figure S1 to S7
Table S1 to S5
References

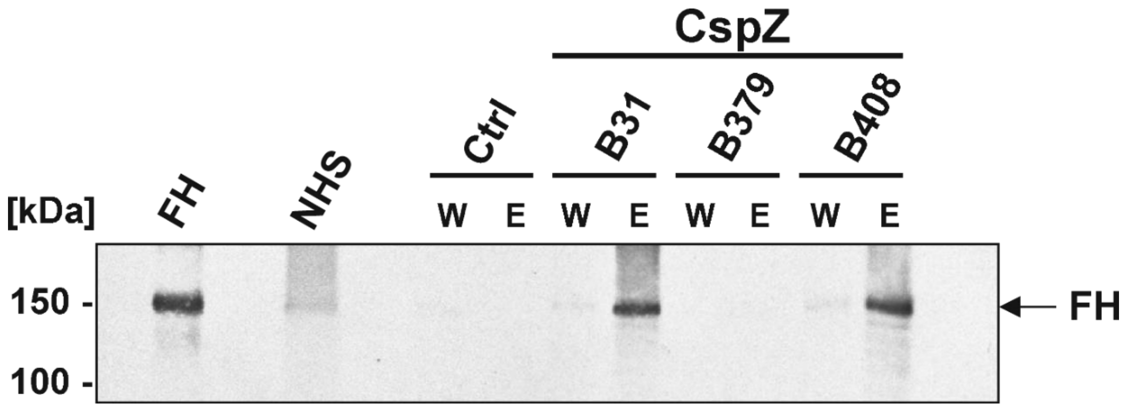

Figure S1. Adsorption assays showed a recombinant CspZ variant-specific human Factor H-binding activity. The indicated CspZ variants immobilized on magnetic particles was incubated in 500µl NHS. Following serum incubation, magnetic particles were washed extensively, and proteins bound were eluted using 0.1 M glycine (pH 2.0). Both the last wash ("W") and the eluate ("E") fractions obtained from each reaction were separated by 10% Tris/tricine SDS-PAGE under non-reducing conditions and transferred to nitrocellulose. Human Factor H was detected by using a goat anti-Factor H antibody. Purified human Factor H (500 ng) and NHS were included as controls. The mobility of the molecular mass standard is indicated on the left, and the location reflecting the molecular weights equivalent to Factor H are shown by arrows in the right.

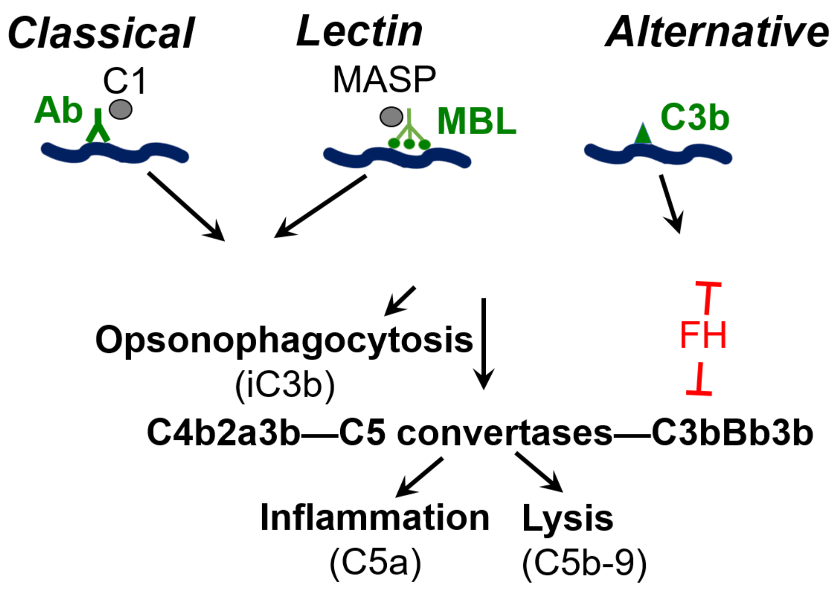

Figure S2. Human complement pathways and Factor H binding-mediated mechanisms to inactivate complement. This figure was adopted from our previous publication, with modifications (49). Complement activation is initiated by one of three pathways, the classical, lectin, and alternative pathways. Classical and lectin pathways are triggered by the antibody-antigen complex whereas the lectin pathway is triggered by the binding of mannose binding lectin (MBL) to microbial carbohydrates. The alternative pathway can be activated by the binding of microbial surface structures to C3b, spontaneously degraded from a serum protein, C3. The activation of complement leads to the deposition of C3-derived products on Lyme borreliae surface converted by the formation of C3 convertases (C4b2a for the classical and alternative pathways and C3bBb for the alternative pathway). The formation of C3 convertases and C3-derived products would lead to the formation of C5 convertases (C4b2a3b for the classical and lectin pathways, C3bBb3b for the alternative pathways), respectively. The formation of C5 convertases results in the formation of a membrane attack complex (C5b-9) on Lyme borreliae surface, leading to cell lysis. One of the complement regulators, Factor H (FH), can inhibit the alternative pathway by recruiting Factor I (FI) to degrade C3b. Lyme borreliae produces Factor H-binding proteins including CspZ to bring Factor H on their surface to prevent the activation of alternative complement pathways on pathogen surface.

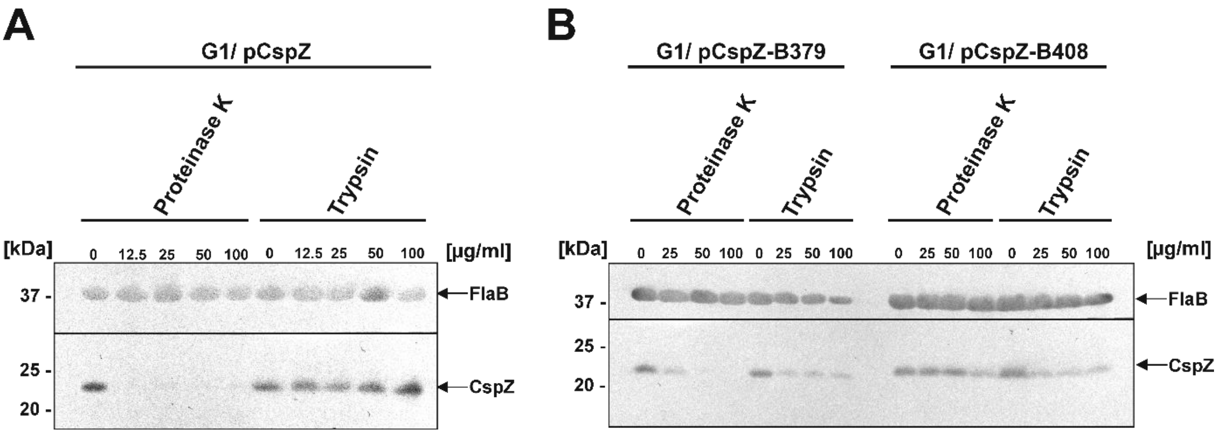

Figure S3. Determination of surface-exposed CspZ from *B. burgdorferi* strains B31, B379 and B408 produced in the gain-of-function *B. garinii* G1. The lysates of indicated *B. garinii* G1-derived strains (5×10^7 treated bacteria) were digested with proteinase K or trypsin at indicated concentration for 2 h. The resulting lysates were applied to a 10% Tris/tricine SDS-PAGE, and CspZ and the periplasmic control protein, FlaB, were identified by Western blotting with a polyclonal mouse antiserum against $CspZ_{B31}$ and the monoclonal antibody L41 1C11 against FlaB of *B. burgdorferi*, respectively. The mobility of the molecular mass standards are indicated on the left, and the location reflecting the molecular weights equivalent to FlaB and CspZ are indicated by arrows at the right.

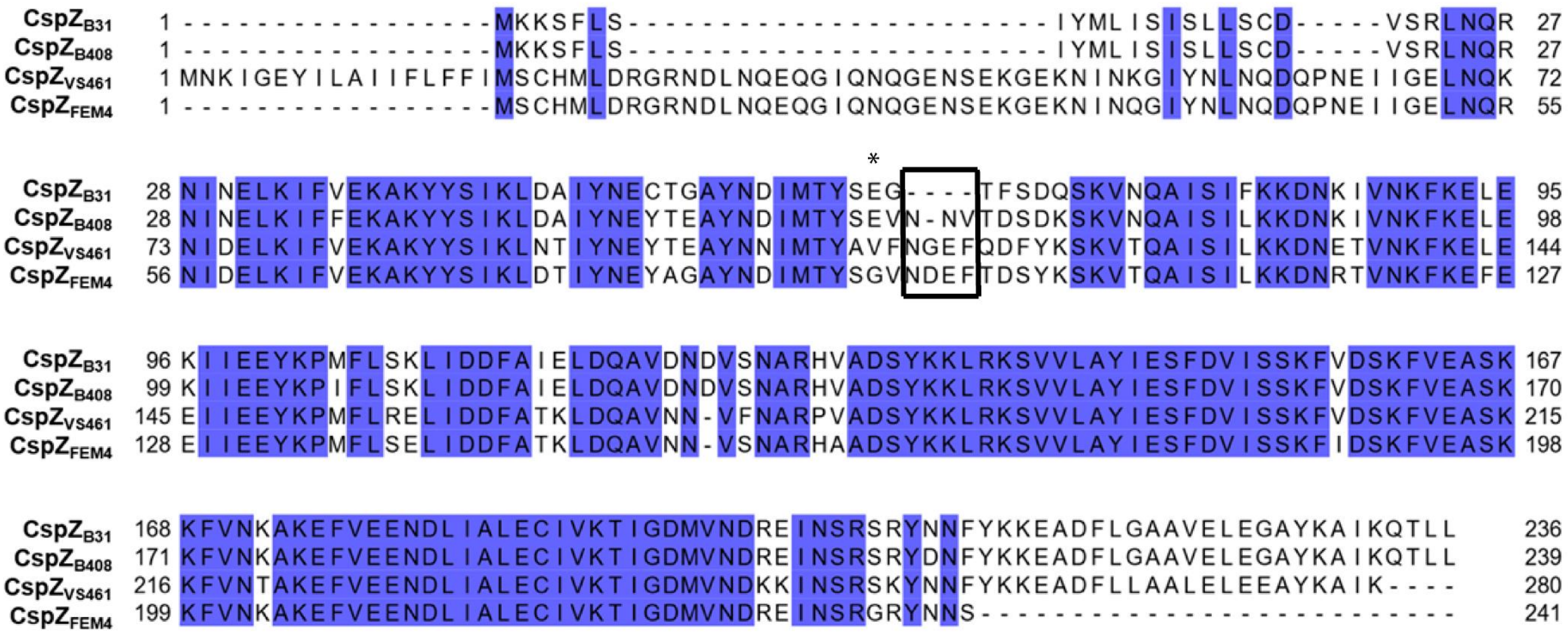

Figure S4. Protein sequence alignment of CspZ variants from *B. burgdorferi* B31 and B408, and *B. afzelii* VS461 and FEM4. CspZ variants from *B. burgdorferi* B31 and B408, and *B. afzelii* VS461 and FEM4, were aligned in tCoffee and visualized with Jalview. The shading indicates complete sequence conservation across variants. The black open box highlights the inserted residues from Asn67 to Val69 of $CspZ_{B408}$. The asterisk indicates the Glu65 site in $CspZ_{B31}$, or equivalent sites in other variants.

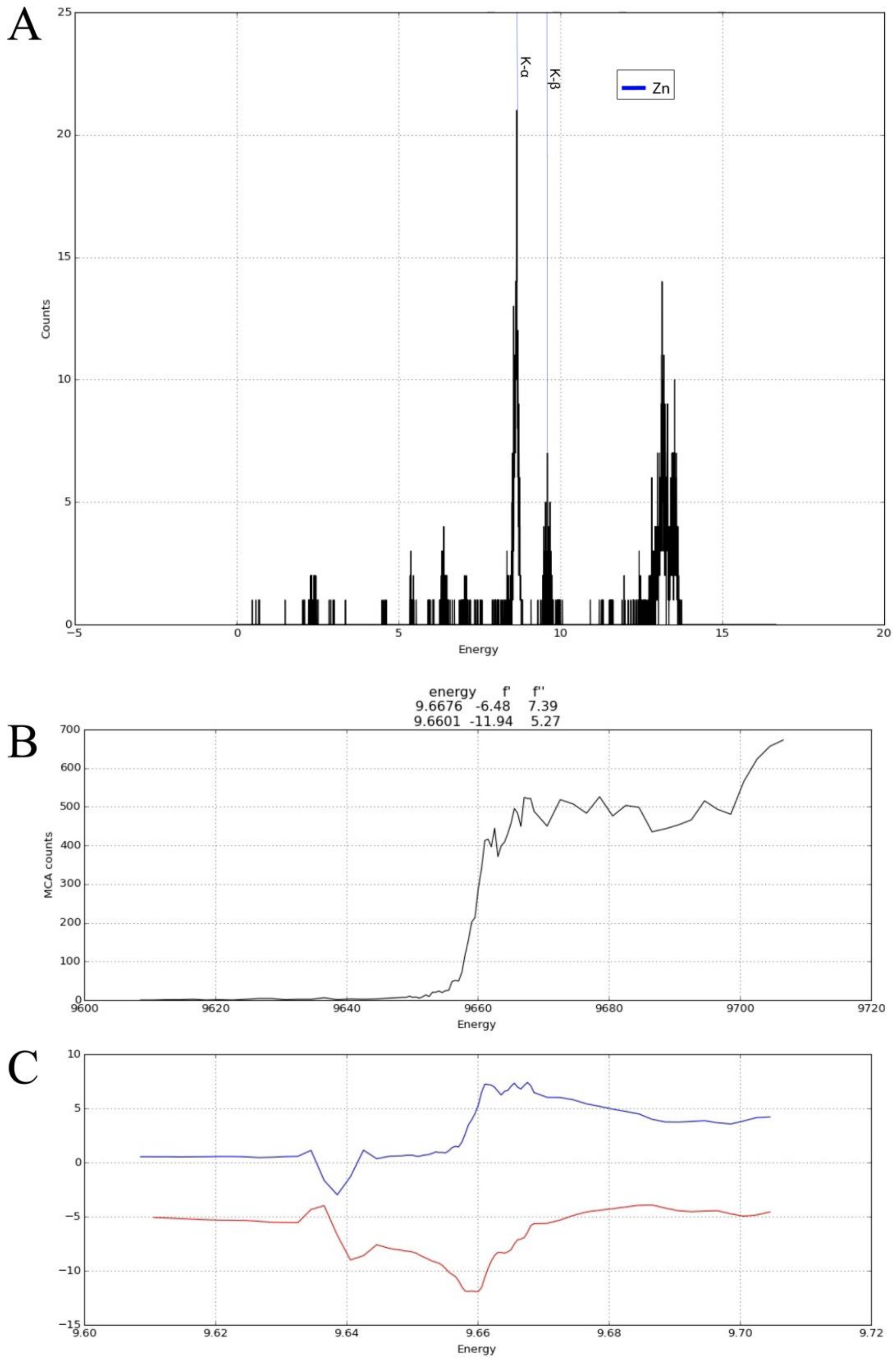

Figure S5. X-ray absorption spectroscopy data for the CspZ$_{B31}$-SCR6-7 complex. (A) X-ray fluorescence (XRF) spectrum, displaying intensity (counts per second) on the y-axis and energy (eV) on the x-axis, revealing a major peak that corresponds to the presence of a zinc in the crystal. (B) An X-ray absorption scan showing the absorption around the Zn *K*-edge matching the characteristic X-ray absorption edge energy for Zn (9.6586 keV). (C) Values for *f′* (red curve) and *f″* (blue curve) around the Zn *K*-edge, plotted against the X-ray energy, used to calculate the inflection point energy and the peak energy around the Zn *K*-edge.

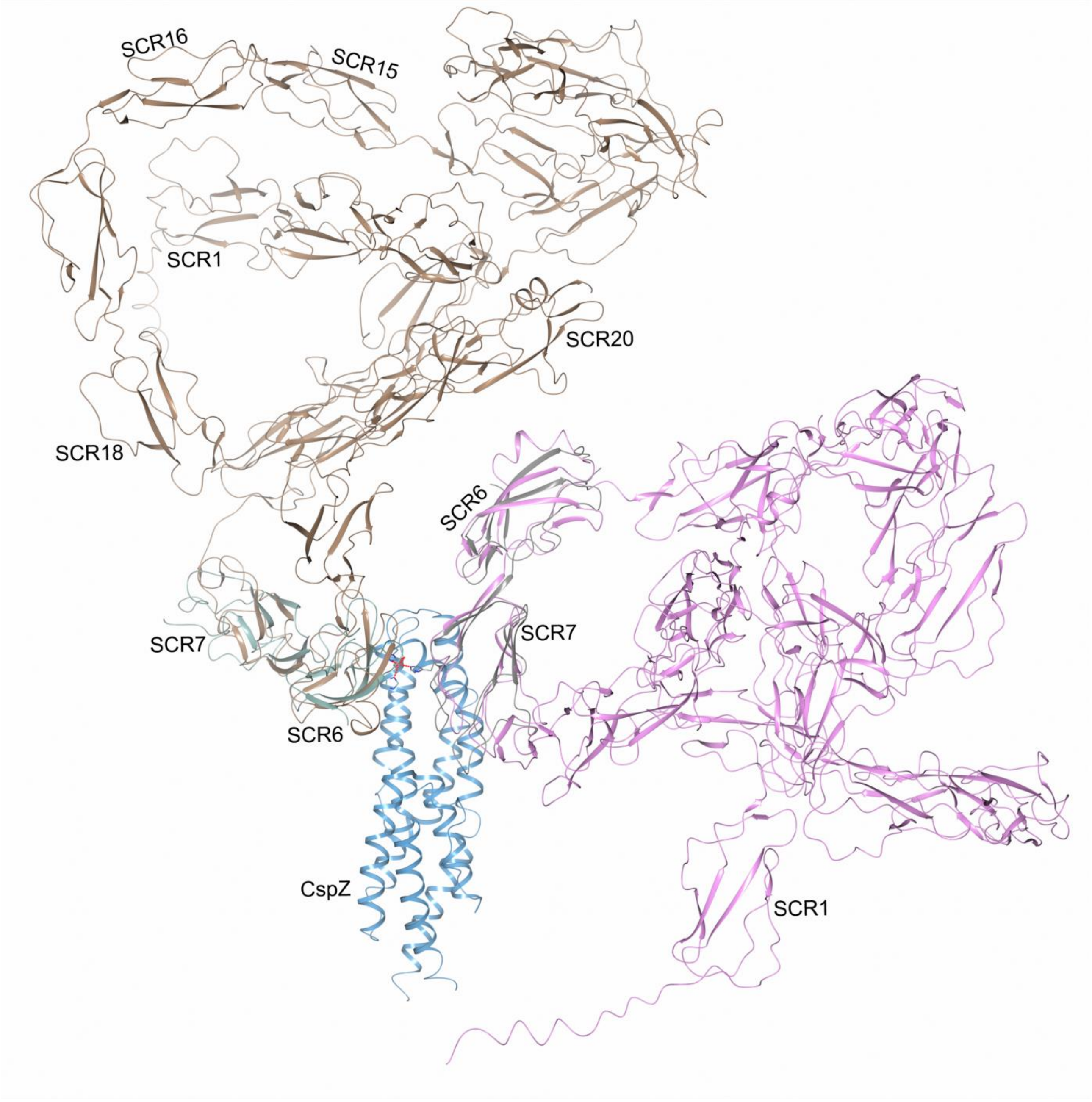

Figure S6. Superimposition of CspZ$_{B31}$ and two FH molecules. The crystal structure of the CspZ$_{B31}$ and human SCR6-7 complex superimposed with the predicted full-length human FH structure. Both SCR6-7 molecules (green and gray, respectively) observed in the crystal structure in complex with CspZ$_{B31}$ (blue) are superimposed with the human FH (brown and pink, respectively). Some of the SCR domains of FH are numbered.

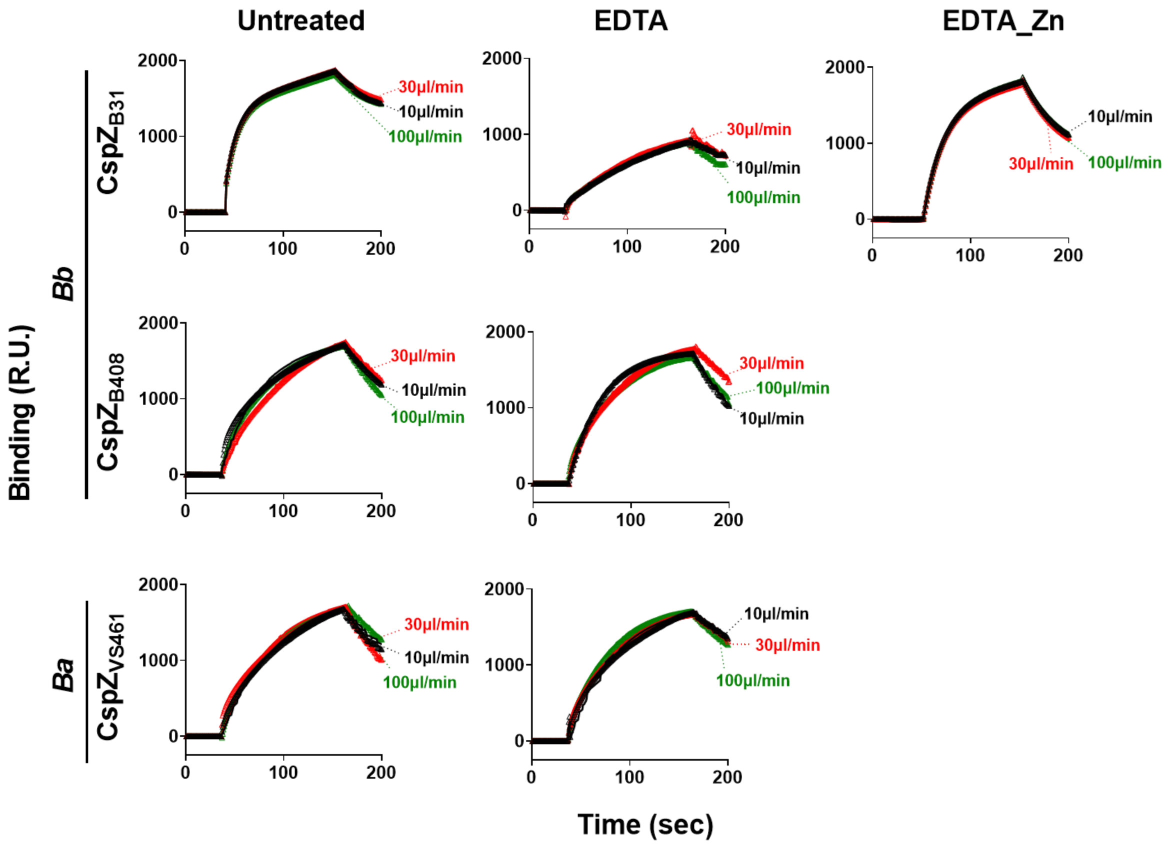

Figure S7. The flow rates do not impact the kinetics of CspZ variants binding to Factor H. The indicated untagged CspZ variants from *B. burgdorferi* (*Bb*), *B. afzelii* (*Ba*) at the concentration of 2µM were treated with 100 µM of EDTA ("EDTA"), followed by dialysis with PBS buffer, or the EDTA-treated, PBS-dialyzed CspZ$_{B31}$ was subsequently treated 100µM of zinc sulfate ("EDTA-Zn"). The indicated CspZ variants under each of the treatment were then flowed in PBS buffer over the chip surface, conjugated with indicated human Factor H, at the flow rates as 10, 30, or 100µl/min. Shown are the representative results of binding measured in response units (R.U.) by SPR from one of three experiments. The k_{on}, k_{off}, and K_D values were determined from the average of all three experiments (**Table S4**).

Table S1. CspZ variants in Lyme borreliae with known human Factor H binding activity and amino acid sequences publicly available

Species	Strain	Accession	References for Factor H binding
B. burgdorferi	B31	NC_001853.1	(26,28,32,33)
	LDP60	FJ911663.1	(28)
	LDP61	FJ911664.1	(28)
	LDP73	FJ911665.1	(28)
	LDS76	FJ911666.1	(28)
	LDP89	FJ911667.1	(28)
	LDS106	FJ911668.1	(28)
	BL206	FJ911669.1	(28)
	B479	FJ911670.1	(28)
	LDP84	FJ911680.1	(28)
	LDP120	FJ911679.1	(28)
	B331	CP017213.1	(28)
	B356	GCA_024733935.1	(28)
	B408	OM643340.1	(28)
	3028	NA[a]	(28)
	JD1	NC_017405.1	(28)
	LP5	NA[a]	(28)
	LW2	EU272845.1	(33,34,37,38)
	ZS7	NC_011781.1	
B. afzelii	VS461	QHO60340.1	(36)
B. spielmanii	A14S	EU272854.1	(38)

[a]The AA sequences were transcribed directly from a figure in the associated reference

Table S2. The strains used in this study.

**Strain or plasmid**	**Genotype or characteristic**	**Source**
Borrelia burgdorferi		
B31-5A4	Clone of *B. burgdorferi* B31 RST type 1, *ospC* type A, isolated from *Ixodes scapularis* in Massachusetts, USA	(76)
B379	Clone of *B. burgdorferi* B379 isolated from Lyme disease patients with erythema migrans in USA. RST Type 2, *ospC* type K	(21)
B408	Clone of *B. burgdorferi* B408 isolated from Lyme disease patients with erythema migrans in USA. RST type 3, *ospC* type I	(21)
Borrelia afzelii		
VS461	Clone of *B. afzelii* and the reference strain isolated from *Ixodes ricinus* in Switzerland	(77)
FEM4	Clone of *B. afzelii* FEM4 isolated from Lyme disease patients with erythema migrans in Germany	(27)
Borrelia garinii		
PBr	Clone of *B. garinii* PBr isolated from CSF of a Lyme disease patient in Germany	(78)
G1	Clone of *B. garinii* G1 isolated from CSF of a Lyme disease patient in Germany	(79)
G1/Vector	G1 carrying plasmid pKFSS1	(80)
G1/pCspZ$_{B31}$	G1 carrying with intact *cspZ*$_{B31}$ driven by the promoter of *cspZ*$_{B31}$	(80)
G1/pCspZ$_{B379}$	G1 carrying with intact *cspZ*$_{B379}$ driven by the promoter of *cspZ*$_{B379}$	(30)
G1/pCspZ$_{B408}$	G1 carrying with intact *cspZ*$_{B408}$ driven by the promoter of *cspZ*$_{B408}$	(30)
Escherichia coli		
DH5α	F- Φ80lacZΔM15 Δ(lacZYA-argF) U169 recA1 endA1 hsdR17(rk-, mk+) phoA	ThermoFisher

	supE44 thi-1 gyrA96 relA1 λ-	
BL21(DE3)	F−, ompT hsdSB (rB− mB−) gal dcm (DE3)	Novagen
BL21(DE3)/pGEX4T2	BL21(DE3) producing GST	(27)
BL21(DE3)/pGEX4T2-$CspZ_{B31}$	BL21(DE3) producing GST-tagged residues 58 to 711 of $CspZ_{B31}$	(26)
BL21(DE3)/pGEX4T2-$CspZ_{B379}$	BL21(DE3) producing GST-tagged residues 58 to 723 of $CspZ_{B379}$	(27)
BL21(DE3)/pGEX4T2-$CspZ_{B408}$	BL21(DE3) producing GST-tagged residues 58 to 720 of $CspZ_{B408}$	(27)
BL21(DE3)/pGEX4T2-$CspZ_{VS461}$	BL21(DE3) producing GST-tagged residues 24 to 280 of $CspZ_{VS461}$	(36)
BL21(DE3)/pGEX4T2-$CspZ_{FEM4}$	BL21(DE3) producing GST-tagged residues 21 to 280 of $CspZ_{FEM4}$	This study
BL21(DE3)/pGEX4T2-$CspZ_{PBr}$	BL21(DE3) producing GST-tagged residues 21 to 280 of $CspZ_{PBr}$	This study
BL21(DE3)/pETm-11-$CspZ_{B31}$	BL21(DE3) producing His-tagged residues 58 to 711 of $CspZ_{B31}$	(31)
Pichia pastoris		
X-33/FH SCR6-7	Wild-type Mut+ Pichia strain for expression of FH SCR6-7 (residues 321 to 444 of human Factor H)	Invitrogen
Plasmids		
pGEX4T2-$CspZ_{FEM4}$	AmpR[a]; pGEX4T2 encoding GST fusion protein residue 21 to 280 of $CspZ_{FEM4}$	This study
pGEX4T2-$CspZ_{PBr}$	AmpR; pGEX4T2 encoding GST fusion protein residue 21 to 280 of $CspZ_{PBr}$	This study

Table S3. Primers used in this study.

Purpose	Primer	Sequence
Generate the plasmid encoding *cspZ*$_{FEM4}$	cspZ$_{\text{FEM4}}$fp	GCGGATCCCATATGTTAGACAGAGGGC
	cspZ$_{\text{FEM4}}$rp	GCGTCGACTTACTTAATAGCTTTATAAGC
Generate the plasmid encoding *cspZ*$_{PBr}$	cspZ$_{\text{PBr}}$fp	GCGGATCCCATATGTTAGACAGAGGGCGA
	cspZ$_{\text{PBr}}$rp	GCGTCGACTTACTTAATAGCTTTATAAGC

Table S4. The kinetic parameters of CspZ variants binding to Factor H under different flow rates.

			----- Surface Plasmon Resonance[b] -----		
**CspZ variant[a]**	**Treatment**	**Flow rate (µl/min)**	**K_D (µM)**	k_{on} ($10^3 s^{-1}M^{-1}$)	k_{off} (s^{-1})
**$CspZ_{B31}$**	**PBS**	**10**	**0.23±0.09**	68.76±4.58	0.016±0.006
		**30**	**0.27±0.13**	63.25±4.52	0.017±0.007
		**100**	**0.25±0.04**	69.16±1.58	0.017±0.002
	**EDTA**	**10**	**3.37±0.23[c]**	4.56±0.22[c]	0.012±0.003[c]
		**30**	**3.73±0.08[c]**	5.79±0.10[c]	0.021±0.004[c]
		**100**	**3.67±0.05[c]**	4.83±0.69[c]	0.017±0.003[c]
	**EDTA-Zn**	**10**	**0.62±0.03**	60.90±3.04	0.037±0.020
		**30**	**0.57±0.01**	61.73±7.35	0.034±0.007
		**100**	**0.66±0.03**	66.61±1.27	0.044±0.010
**$CspZ_{B408}$**	**PBS**	**10**	**0.55±0.23**	16.54±3.07	0.008±0.002
		**30**	**0.53±0.17**	13.56±1.99	0.007±0.001
		**100**	**0.54±0.20**	13.16±2.68	0.006±0.002
	**EDTA**	**10**	**0.48±0.19**	15.66±2.36	0.007±0.002
		**30**	**0.48±0.07**	13.64±9.71	0.006±0.001
		**100**	**0.50±0.09**	16.71±3.49	0.008±0.003
**$CspZ_{VS461}$**	**PBS**	**10**	**0.37±0.03**	10.81±4.23	0.004±0.001
		**30**	**0.38±0.07**	10.82±5.63	0.004±0.002
		**100**	**0.34±0.17**	10.50±2.55	0.003±0.009
	**EDTA**	**10**	**0.48±0.23**	10.66±2.50	0.004±0.001
		**30**	**0.43±0.20**	9.16±1.56	0.003±0.001
		**100**	**0.11±0.11**	11.09±2.93	0.004±0.004

All values represent the mean ± standard deviation of three experiments
[a]The CspZ variants applied to this study were 1µM
[b]Determined using untagged CspZ proteins
[c]Because the binding of EDTA treated $CspZ_{B31}$ to human Factor H is weak, the determined values should be considered estimates.

Table S5. Data processing, refinement, and validation statistics of crystal structures.

[a]Values in parentheses are for the highest resolution bin.

**Dataset**	**$CspZ_{B31}$-SCR6-7**
X-ray diffraction data	
PDB entry	9F7I
Beamline	BESSY II beamline 14.1
Space group	$P22_12_1$
a, b, c (Å)	44.66, 67.10, 143.60
α, β, γ (°)	90.0, 90.0, 90.0
Wavelength (Å)	0.91840
Resolution (Å)	49.02-2.85
Highest resolution bin (Å)	2.92-2.85
No. of reflections	135499
No. of unique reflections	10673
Completeness (%)	100.0 (100.0)[a]
R_{merge}	0.151 (0.437)
$CC_{1/2}$	0.995 (0.949)
I/σ (I)	11.5 (4.6)
Multiplicity	12.7 (12.1)
Refinement	
R_{work}	0.201 (0.246)
R_{free}	0.260 (0.347)
Average B-factor (Å^2)	
Overall	52.5
From Wilson plot	47.1
No. of atoms	
Protein	2670
Water	62
RMS deviations from ideal	
Bond lengths (Å)	0.008
Bond angles (°)	1.647
MolProbity score	2.30
Ramachandran outliers (%)	
Residues in most favored regions (%)	92.62
Residues in allowed regions (%)	7.38
Outliers (%)	0

References

21. Lin, Y. P., Diuk-Wasser, M. A., Stevenson, B., and Kraiczy, P. (2020) Complement Evasion Contributes to Lyme Borreliae-Host Associations. *Trends in parasitology* **36**, 634-645

26. Marcinkiewicz, A. L., Dupuis, A. P., 2nd, Zamba-Campero, M., Nowak, N., Kraiczy, P., Ram, S., Kramer, L. D., and Lin, Y. P. (2019) Blood treatment of Lyme borreliae demonstrates the mechanism of CspZ-mediated complement evasion to promote systemic infection in vertebrate hosts. *Cellular microbiology* **21**, e12998

27. Marcinkiewicz, A. L., Brangulis, K., Dupuis, A. P., 2nd, Hart, T. M., Zamba-Campero, M., Nowak, T. A., Stout, J. L., Akopjana, I., Kazaks, A., Bogans, J., Ciota, A. T., Kraiczy, P., Kolokotronis, S. O., and Lin, Y. P. (2023) Structural evolution of an immune evasion determinant shapes pathogen host tropism. *Proceedings of the National Academy of Sciences of the United States of America* **120**, e2301549120

28. Rogers, E. A., Abdunnur, S. V., McDowell, J. V., and Marconi, R. T. (2009) Comparative analysis of the properties and ligand binding characteristics of CspZ, a factor H binding protein, derived from *Borrelia burgdorferi* isolates of human origin. *Infection and immunity* **77**, 4396-4405

30. Nowak, T. A., Lown, L. A., Marcinkiewicz, A. L., Surth, V., Kraiczy, P., Burke, R., and Lin, Y. P. (2023) Outer surface protein E (OspE) mediates *Borrelia burgdorferi* sensu stricto strain-specific complement evasion in the eastern fence lizard, Sceloporus undulatus. *Ticks and tick-borne diseases* **14**, 102081

31. Brangulis, K., Petrovskis, I., Kazaks, A., Bogans, J., Otikovs, M., Jaudzems, K., Ranka, R., and Tars, K. (2014) Structural characterization of CspZ, a complement regulator factor H and FHL-1 binding protein from *Borrelia burgdorferi*. *The FEBS journal* **281**, 2613-2622

33. Marcinkiewicz, A. L., Lieknina, I., Kotelovica, S., Yang, X., Kraiczy, P., Pal, U., Lin, Y. P., and Tars, K. (2018) Eliminating Factor H-Binding Activity of *Borrelia burgdorferi* CspZ Combined with Virus-Like Particle Conjugation Enhances Its Efficacy as a Lyme Disease Vaccine. *Frontiers in immunology* **9**, 181

34. Muhleip, J. J., Lin, Y. P., and Kraiczy, P. (2018) Further Insights Into the Interaction of Human and Animal Complement Regulator Factor H With Viable Lyme Disease Spirochetes. *Front Vet Sci* **5**, 346

35. Kraiczy, P., Skerka, C., Brade, V., and Zipfel, P. F. (2001) Further characterization of complement regulator-acquiring surface proteins of *Borrelia burgdorferi*. *Infection and immunity* **69**, 7800-7809

37. Marcinkiewicz, A. L., Lieknina, I., Yang, X., Lederman, P. L., Hart, T. M., Yates, J., Chen, W. H., Bottazzi, M. E., Mantis, N. J., Kraiczy, P., Pal, U., Tars, K., and Lin, Y. P. (2020) The Factor H-Binding Site of CspZ as a Protective Target against Multistrain, Tick-Transmitted Lyme Disease. *Infection and immunity* **88**

38. Kraiczy, P., Skerka, C., Kirschfink, M., Brade, V., and Zipfel, P. F. (2001) Immune evasion of *Borrelia burgdorferi* by acquisition of human complement regulators FHL-1/reconectin and Factor H. *European journal of immunology* **31**, 1674-1684

39. Herzberger, P., Siegel, C., Skerka, C., Fingerle, V., Schulte-Spechtel, U., van Dam, A., Wilske, B., Brade, V., Zipfel, P. F., Wallich, R., and Kraiczy, P. (2007) Human

pathogenic *Borrelia spielmanii* sp. nov. resists complement-mediated killing by direct binding of immune regulators factor H and factor H-like protein 1. *Infection and immunity* **75**, 4817-4825

50. Tufts, D. M., Hart, T. M., Chen, G. F., Kolokotronis, S. O., Diuk-Wasser, M. A., and Lin, Y. P. (2019) Outer surface protein polymorphisms linked to host-spirochete association in Lyme borreliae. *Molecular microbiology* **111**, 868-882

76. Purser, J. E., Lawrenz, M. B., Caimano, M. J., Howell, J. K., Radolf, J. D., and Norris, S. J. (2003) A plasmid-encoded nicotinamidase (PncA) is essential for infectivity of *Borrelia burgdorferi* in a mammalian host. *Molecular microbiology* **48**, 753-764

77. Baranton, G., Postic, D., Saint Girons, I., Boerlin, P., Piffaretti, J. C., Assous, M., and Grimont, P. A. (1992) Delineation of *Borrelia burgdorferi* sensu stricto, *Borrelia garinii* sp. nov., and group VS461 associated with Lyme borreliosis. *International journal of systematic bacteriology* **42**, 378-383

78. Wilske, B., Preac-Mursic, V., Gobel, U. B., Graf, B., Jauris, S., Soutschek, E., Schwab, E., and Zumstein, G. (1993) An OspA serotyping system for *Borrelia burgdorferi* based on reactivity with monoclonal antibodies and OspA sequence analysis. *Journal of clinical microbiology* **31**, 340-350

79. Kraiczy, P., Hunfeld, K. P., Breitner-Ruddock, S., Wurzner, R., Acker, G., and Brade, V. (2000) Comparison of two laboratory methods for the determination of serum resistance in *Borrelia burgdorferi* isolates. *Immunobiology* **201**, 406-419

80. Kraiczy, P., Seling, A., Brissette, C. A., Rossmann, E., Hunfeld, K. P., Bykowski, T., Burns, L. H., Troese, M. J., Cooley, A. E., Miller, J. C., Brade, V., Wallich, R., Casjens, S., and Stevenson, B. (2008) *Borrelia burgdorferi* complement regulator-acquiring surface protein 2 (CspZ) as a serological marker of human Lyme disease. *Clinical and vaccine immunology : CVI* **15**, 484-491
